# Protective Effects of Engineered *Lactobacillus crispatus* on Intrauterine Adhesions in Mice *via* Delivering CXCL12

**DOI:** 10.3389/fimmu.2022.905876

**Published:** 2022-06-06

**Authors:** Yao Kong, Zhaoxia Liu, Qin Xiao, Fei Wu, Lijuan Hu, Xiaorong Deng, Tingtao Chen

**Affiliations:** ^1^ Department of Obstetrics and Gynecology, The Second Affiliated Hospital of Nanchang University, Nanchang, China; ^2^ Department of Gastrointestinal Surgery, The Second Affiliated Hospital of Nanchang University, Nanchang, China; ^3^ National Engineering Research Center for Bioengineering Drugs and The Technologies, Institute of Translational Medicine, Nanchang University, Nanchang, China

**Keywords:** *Lactobacillus crispatus*, mCXCL12, pMG36e, intrauterine adhesions, high-throughput sequencing

## Abstract

Endometrial injury is the main cause of intrauterine adhesions (IUA), and there is currently no effective prevention and treatment. Immune cells play an important role in damage repair by sensing the change in the microenvironment. Exogenous CXCL12 can promote tissue regeneration and repair by recruiting immune cells, but its effect and possible mechanism on endometrial regeneration and repair have not been reported. In the present study, we constructed an engineered a *Lactobacillus crispatus* strain by transforming a pMG36e plasmid carrying a CXCL12 gene into the bacterium, and developed two animal models, the intrauterine adhesion mice with or without diabetes to evaluate the positive effects of this strain on the prevention of IUA after accepting intrauterine surgery in normal and diabetic mice. The results showed that vaginal application of *L. crispatus*-pMG36e-mCXCL12 strains significantly diminished the levels of pro-inflammatory factors interleukin-1β (IL-1β) and tumour necrosis factor-α (TNF-α) in serum and uterine tissues of IUA mice, and resulted in the inhibition of the inflammatory (toll-like receptor 4/nuclear factor-κb, TLR4/NF-κB) and fibrotic (transforming growth factor-β1/smads, TGF-β1/Smads) signalling pathways in the uterine tissues. The high-throughput sequencing results further indicated that treatment with *L. crispatus*-pMG36e-mCXCL12 strains greatly increased the abundance of *Lactobacillus* spp. and reduced that of the pathogenic *Klebsiella* spp. in IUA mice. Furthermore, among intrauterine adhesion mice with diabetes, we obtained similar results to non-diabetic mice, that is, *L.crispatus*-pMG36e-mCXCL12 significantly improved fibrosis and inflammation in the uterine cavity of diabetic mice, and restored the vaginal microbiota balance in diabetic mice. Therefore, we speculated that vaginal administration of *L. crispatus*-pMG36e-mCXCL12 strains can effectively alleviate intrauterine adhesions by restoring the microbial balance and reducing inflammation and fibrosis caused by surgery.

## Introduction

Intrauterine adhesion (IUA) refers to partial or complete occlusion of the uterine cavity after the endometrial basal layer is damaged (1). Statistics show that the incidence rate of IUA caused by repeated abortions, curettage and other intrauterine operations in China is as high as 25-30% (2), making it the major cause of decreased menstrual flow and secondary infertility (3). Currently, transcervical resection of adhesion (TCRA) is the main therapy for IUA. Although postoperative adjuvant therapy, such as placing intrauterine devices or hydrogel, and sequentially providing oestrogen and progesterone, have been used to prevent re-adhesion (4). However, the high treatment cost, displacement, or even shedding of the physical barrier, as well as the side effects of drug therapy (5), have led to unsatisfactory treatment effects. Therefore, it is particularly urgent to find an effective and ideal method to prevent adhesions.

The process of injury and repair mainly includes three stages: inflammatory reaction, tissue formation and tissue remodeling (6). Most injuries are incompletely repaired, frequently leading to the formation of hypertrophic scars (7). As a common iatrogenic disease, IUA is essentially an inflammatory and fibrotic disease. The operation carried out in the uterine cavity destroys the endometrial barrier, and the subsequent continuous infection and inflammation will inhibit the regeneration of the damaged endometrium, then leading to endometrial fibrosis and intrauterine adhesions (8). It is well-known that the mobilization and proliferation of cells within the body is an important procedure to promote the repair of damaged tissue (9). Studies have found that CXCL12/CXCR4 axis is stimulated under certain pathological conditions, such as hypoxia, inflammation, ischemia, cancer and autoimmune diseases, and CXCL12/CXCR4 axis is involved in mediating cell chemotaxis and metastasis, promoting immune regulation and may finally inducing angiogenesis (10, 11). In general, the hypoxic environment of endometrium is a normal physiological phenomenon in the premenstrual period, and it is an important regulatory factor for endometrial tissue angiogenesis and tissue remodeling (12). Research on the function of CXCL12 in human endometrial tissue suggests that it plays an important role in endometrial hyperplasia and embryo implantation (11), but endogenous CXCL12 cannot meet the needs of tissue repair in the condition of severe tissue injury, making it necessary to introduce exogenous CXCL12. In addition, studies have shown that ischemia or hyperglycemia damage in local immune response is more intense, which makes the damage repair time longer (13). Moreover, in long-term clinical practice, we found that women with diabetes are more likely to develop intrauterine adhesions after intrauterine surgery.

In recent years, researchers have found that the repair of the intestinal mucosal injury is regulated by local complex neuronal networks, immune cells, endothelial cells and the extracellular matrix of the mucosal lamina propria (14). In addition, it is also closely related to intestinal microbiota (15). Intestinal probiotics like *Bifidobacterium* and *Lactobacillus* have been widely used in the repair of the intestinal mucosal barriers by inhibiting the overgrowth of potentially pathogenic bacteria, promoting the secretion of immune proteins by cells around the intestine, and modulating systemic immunity (16, 17). Currently, the application of probiotics has become an important research area. There is increasing evidence that the vaginal microbiota plays a critical role in female reproductive tract health, and *Lactobacillus* is regarded as the most dominant physiological bacterial genus, impeding the over proliferation of pathogenic bacteria by decomposing glycogen in vaginal epithelial cells into lactic acid (18). In addition, it can inhibit the overgrowth of pathogenic bacteria by producing several antimicrobial secondary metabolites (hydrogen peroxide, bacteriocins, and biosurfactants) (19). Some *Lactobacilli* can inhibit the adhesion of pathogenic bacteria by competitively binding to receptors on the host surface or by destroying the biofilms of pathogenic bacteria (20). As the dominant *Lactobacillus* in the vaginal tract, *L. crispatus* belongs to the food-grade bacteria: it shows rapid growth, highly safe, good stability and non-endotoxin productiion, this is why it is often used as a probiotic for the treatment of bacterial vaginosis and other diseases (21).

In the present study, we constructed an engineered *L. crispatus* which could express mCXCL12, with a net expression level of about 94 pg/mL detected by ELISA. To increase the reliability of the engineered commensal bacteria, we further conducted the stability test of the recombinant *L. crispatus-*pMG36e-mCXCL12 cells. To examine the effects of this engineered strain at intact animal level, we constructed two intrauterine adhesion mouse models, one with diabetes and one without diabetes, in consideration of women with diabetes are more likely to develop intrauterine adhesions after intrauterine surgery. We further compared the preventive effects of *L.crispatus*-pMG36e-mCXCL12 on the formation of intrauterine adhesion in normal mice and mice with diabetes to explore the potential of *L. crispatus*-pMG36e-mCXCL12 being used as a preventive agent against IUA.

## Methods

### Construction of Expression Strains and Protein Expression

To generate *L. crispatus*-pMG36e-mCXCL12, the DNA sequence for murine CXCL12 (NM_021704.3) was synthetically produced by Sangon Biological Engineering Co., Ltd (Shanghai, China), and inserted into pMG36e (a prokaryotic expression vector) by restriction enzyme-mediated cloning, followed by the formation of the pMG36e-mCXCL12 plasmid. Lactobacillus crispatus Lcr-MH175 (number CGMCC 15938, identified by Harbin Meihua Biotechnology Co., Ltd., Harbin, Heilongjiang, China) was electrically transformed with the above plasmid to generate the *L. crispatus*-pMG36e-mCXCL12 strain. Both *L. crispatus* and *L. crispatus*-pMG36e-mCXCL12 strains were incubated overnight without antibiotics and with erythromycin (200 μg/ml) from MRS (Hopebio, Qingdao, China), respectively. The overnight cultures were re-inoculated in fresh medium at a dilution of 1:100. After 24 h, samples were centrifuged (>3,000 × rpm, 6 min), and the pellet and supernatant were separated. Bacteria were resuspended with PBS, pulverized with ultrasound, and then centrifuged at 13,000 RPM for 15 min, and the supernatant was taken for detection. Secreted CXCL12 was measured using the ELISA Kit for Stromal Cell-Derived Factor 1 (Cloud-Clone, WuHan, China).

### *In Vitro* Analysis of Plasmid Stability

Single colonies of plasmid strain *L.crispatus*-pMG36e-mCXCL12 were selected and inoculated in MRS (Hopebio, Qingdao, China) liquid medium containing erythromycin. On the next day, the plasmid strains were inoculated in MRS liquid medium containing erythromycin and without erythromycin at a ratio of 1:100 for 24 hours. After that, the new MRS liquid medium containing erythromycin and without erythromycin was used for subculture every day until 30 days. At the same time, take the corresponding bacterial solution every 3 days for plate scribing (non-resistant MRS plate), select 100 single plasmid strain sites randomly and plant them on MRS plate containing erythromycin, culture them at 37°C for 16-24 h, and calculate the number of growing colonies until the end of the experiment. Based on the growth of 100 colonies planted on erythromycin plates, the genetic stability rate of *L.crispatus*-pMG36e-mCXCL12 recombinant plasmid was calculated.

### Animal Models and Treatments

Seventy female BALB/c mice (6 weeks, 25–30 g) were provided by Hunan Si Lake King of Experimental Animal Co., Ltd. The mice were raised in specific pathogen-free cages in the animal house of the Institute of Translational Medicine of Nanchang University under standard conditions (humidity 51–13%, temperature 23 ± 3°C, light-dark cycle 12/12), and food and water were supplied for ad libitum. The mice were adaptively fed for 7 days before the start of the experiment and discretionarily divided into seven groups: (i) Control group (C, n = 10), only the abdominal wall was cut and sutured without other surgical treatment, and then treated with an absorbable collagen sponge (ACS) containing physiological saline solution *via* vagina (administration for 7 consecutive days, operation day 1 to day 7). (ii) Model group 1 (M, n = 10), laparotomy was used to construct a model of intrauterine adhesion mice without diabetes; (iii) *L. crispatus*-treated intrauterine adhesion mice without diabetes (NT, n = 10), treated with an ACS containing *L. crispatus* (10^8^ CFU/ml) *via* vagina (administration for 7 consecutive days, operation day 1 to day 7); (iv) *L. crispatus*-pMG36e-mCXCL12-treated intrauterine adhesion mice without diabetes (ET, n = 10), treated with an ACS containing *L. crispatus*-pMG36e-mCXCL12 (10^8^ CFU/ml) *via* vagina (administration for 7 consecutive days, operation day 1 to day 7). (v) Model of intrauterine adhesion mice with diabetes (DM, n = 10). After constructing the type I diabetes model, laparotomy was carried out to establish a mouse uterine adhesion model. (vi) *L. crispatus*-treated intrauterine adhesion mice with diabetes group (NDT, n = 10), then treated with an ACS containing *L. crispatus* (10^8^ CFU/ml) *via* vagina (administration for 7 consecutive days, operation day 1 to day 7); (vii) *L. crispatus*-pMG36e-mCXCL12-treated intrauterine adhesion mice with diabetes group (EDT, n = 10), treated with an ACS containing *L. crispatus*-pMG36e-mCXCL12 (10^8^ CFU/ml) *via* vagina (administration for 7 consecutive days, operation day 1 to day 7) (22).

Animal experiments were performed in a sterile environment using the laparotomy method (23). All animals were fasted for 12 hours before surgery. Animals were anesthetized by intraperitoneal injection of 1% sodium pentobarbital (40 mg/kg; Cat# B1202-005; Fluka). After anaesthesia, the abdomen was shaved, sterilization with povidone-iodine (Cat# MDS093904; Medline Industries) and covered with sterile surgical towels. Laparotomy was performed by making an incision with a diameter of about 1 cm in the centre of the lower abdomen and clamping the fallopian tube ends of the uterus with straight forceps, dragging out the incision to fully expose the uterine horns on both sides. A transverse incision was made with ophthalmological scissors at 0.5 cm from the end of the uterus near the end of the fallopian tube. The length of the incision was about 1/2 of the diameter of the uterus. We then bend the micro-medicine spoon (diameter 3 mm) into the uterine cavity and repeatedly scraped the endometrium, avoiding puncturing the uterine wall. After suturing the uterine incision, we closed the abdomen and sutured the skin with zero thread. After surgery, all mice were transferred to separate cages according to groups. In addition, weight-matched 8-week-old female mice were treated with STZ (Cat# B1202-005; Fluka) according to the animal models of diabetic complications consortium (AMDCC) protocol. STZ was freshly dissolved in sterile saline and injected intraperitoneally into the mice. Mice treated with 50 mg/kg STZ were fasted for 12 hours prior to STZ induction and injected for 5 consecutive days. Blood glucose was measured in each group of mice in the fed state, and only mice with blood glucose concentrations greater than 400 mg/dl in the fed state were used as diabetics in each group of experiments (24).

The study commenced on October 15, 2018, at the Institute of Translational Medicine of Nanchang University after approval by the Ethical Committee of Nanchang Royo Biotech Co., Ltd (reference number RYE2018121801). All experiments were conducted in accordance with the established guidelines.

### Sample Collection

After the administration was completed, under the premise of aseptic operation, 50 μL of isotonic NaCl solution was aspirated with a micropipette, and the vagina of each mouse was repeatedly washed 8 times; the washings were stored in 50% sterilised glycerine. Subsequently, all animals were euthanized with an overdose of sodium pentobarbital (100 mg/kg; Cat# B1202-005; Fluka). Venous blood was obtained from the eye socket vein and centrifuged at 1,000 x g for 20 min at 4°C. The abdomen was immediately incised to separate the uterus and the vagina, and all tissues were collected and preserved in 4% paraformaldehyde in preparation for hematoxylin and eosin (H&E) staining and for Masson staining. The uteri of the remaining mice were collected for Western blot analysis and reverse transcription-quantitative polymerase chain reaction (RT-qPCR) analysis. All samples were stored at -80°C for future use.

### H&E and Masson Staining

The uterine tissue of each group was fixed in 4% (v/v) paraformaldehyde for 24 hours, and then washed with water and dehydrated with increasing concentrations of alcohol, reacted with the addition of various concentrations of xylene, embedded in paraffin and cut into sections of 5–6 µm in thickness; subsequently, H&E and Masson staining were subsequently performed.

### Western Blotting

Uterine tissues were clipped and lysed in RIPA lysis buffer (Solarbio, Beijing, China; Cat# R0010) for 30 min and centrifuged at 13,000 rpm for 10 min at 4°C. Protein concentration was determined and then separated by polyacrylamide gel electrophoresis using 10–12% SDS-PAGE gels. Proteins were transferred to polyvinylidene difluoride membranes, and then incubated with 5% bovine serum albumin in Tris-buffered saline for 2 hours at room temperature to block non-specific binding. The membranes were then incubated overnight at 4°C with the following primary antibodies: rabbit anti-β-actin, mouse anti TLR4, rabbit anti-NF-κB, rabbit anti-phosphorylated-NF-kB, rabbit anti-TGF-β1, rabbit anti p-Smad2, rabbit anti-Smad2, rabbit anti-p-Smad3, rabbit anti-Smad3, rabbit anti-MMP9. After being washed three times, samples were incubated with goat anti-rabbit secondary antibody or goat anti-mouse secondary antibody for 60 min at RT. Finally, specific proteins were detected using enhanced chemiluminescence (ECL Western blot kit; CwbioTech; Cat# CW0049S).

### Measurement of Cytokines

Venous blood was obtained from the eye socket and centrifuged at 1,000 x g for 20 min at 4°C. Cytokine concentrations in the serum were measured using ELISA kits for IL-1β (Cat# SEA563Mu; mouse; Cloud-Clone Crop; sensitivity range: 15.6–1,000 pg/mL; concentration range used for generating calibration curves: 1,000, 500, 250, 125, 62.5, 31.2, 15.6 and 0 pg/mL) and TNF-α (Cat# SEA133Mu; mouse; Cloud-Clone Crop; sensitivity range: 15.6–1,000 pg/mL; concentrations used for generating calibration curves: 1,000, 500, 250, 125, 62.5, 31.2, 15.6 and 0 pg/mL) according to the manufacturer’s protocols.

### Reverse Transcription-Quantitative Polymerase Chain Reaction (RT-qPCR) Assay

Total RNA from uterine tissues was extracted with Trizol reagent (Gibco BRL; Thermo Fisher Scientific) according to the manufacturer’s protocol. The RNA isolated from each sample was then reverse transcribed into cDNA using the PrimeScript™ RT reagent Kit with gDNA Eraser (Takara Biotechnology Co., Ltd.). The reaction conditions for reverse transcription were as follows: genomic DNA was removed at 42°C for 2 min, reverse transcription at 37°C for 15 min, and reverse transcriptase was inactivated at 85°C for 5 s. Next, Quantitative real-time PCR was performed using a 7500HT fast real-time PCR system (ABI; Thermo Fisher Scientifc, Inc.). The amplification conditions were as follows: pre-denaturation at 95°C for 30 s, followed by PCR reaction for 40 cycles, denaturation at 95°C for 5 s, and annealing at 60°C for 30 s. The relative expression levels were calculated using the 2^−deltadeltaCT^Cq method. The primers used for the RT-qPCR analysis are presented in **Table S1**.

### DNA Extraction and High-Throughput Sequencing

Regarding the extraction of microbial DNA, vaginal secretion samples were collected from groups C (n=8), M (n=7), NT (n=7), ET (n=8), DM (n=6), NDT (n=7), and EDT (n=7). The bead-beating method was combined with genomic DNA kits (Tiangen Biotech Co., Ltd.), followed by measurement of the concentration and quality of purified DNA using a spectrophotometer (NanoDrop; Thermo Fisher Scientifc, Inc.). Further, the 515F/806R primers (515F, 5’-GCACCTAAYTGGGYDTAAAGNG-3’; 806R, 5’-TACNVGGGTATCTAATCC-3’) were used to amplify the V4 region of the 16S rDNA genes in each sample. These PCR products were sequenced using the IlluminaHiSeq 2000 platform (GenBank accession number PRJNA728394).

### Data Analysis

To analyse the high-throughput sequencing data, QIIME (Quantitative Insights Into Microbial Ecology, v1.8.0, http://qiime.org/) (25), the FLASH software (v. 1.2.7, http://ccb.jhu.edu/software/FLASH/), the UCLUST software package and R software were used to compare the α diversity within a sample and β diversity among samples. Microsoft Excel and GraphPad Prism 8 (https://www.graphpad.com/) were used for statistical analyses. Data were expressed as mean ± standard deviation (SD). Student’s t-test and one-way or two-way ANOVA analysis of variance were used for statistical analysis. The results were presented as the mean ± standard deviation (SD), and Student’s t-test was used to compare differences between two groups, while one-way or two-way ANOVA was used to compare differences between multiple groups. For continuous variables that met the requirements of normal distribution, such as age, the data are reported as mean ± SD. Otherwise, they are expressed as quartiles.

## Results

### *crispatus*-pMG36e-mCXCL12 Prevented Intrauterine Adhesions After Intrauterine Surgery in Mice by Inhibiting the Formation of Fibrosis and Inflammation

**Figure 1:**
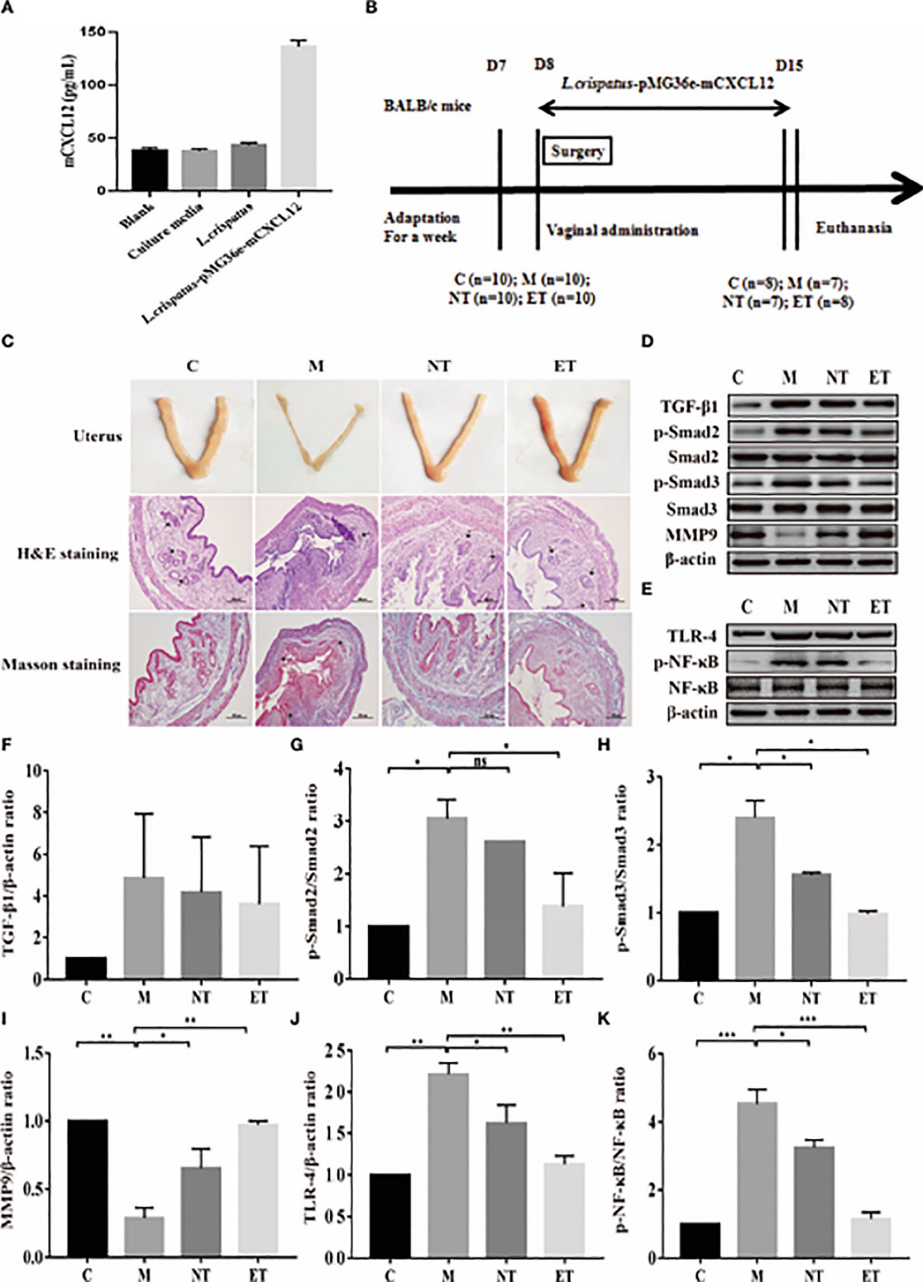
Effect of *L. crispatus*-pMG36e-mCXCL12 on the expression of proteins associated with the TGF-β1/Smads and TLR4/NF-κB signalling pathway in intrauterine adhesion mice. **(A)** mCXCL12 *in-vitro* bacterial expression. **(B)** Experimental scheme to evaluate the effects of *L. crispatus*-pMG36emCXCL12 on the prevention of intrauterine adhesion in mice. **(C)** H&E staining was used to observe uterine inflammation, and Masson staining was used to observe collagen deposition in the uterine tissue of intrauterine adhesion mice (magnification: × 100). **(D)** Expression of fibrotic-related proteins in the uterine tissue of intrauterine adhesion mice. **(E)** Expression of inflammatory-related proteins in the uterine tissue of intrauterine adhesion mice. Effect of *L. crispatus*-pMG36e-mCXCL12 on fibrotic-related TGF-β1 **(F)**, p-Smad2/Smad2 **(G)**, p-Smad3/Smad3 **(H)**, MMP-9 **(I)** proteins in the uterine tissue of intrauterine adhesion mice. Effect of *L. crispatus*-pMG36e-mCXCL12 on inflammatory-related TLR4 **(J)**, p-NF-κB/NF-κB **(K)** proteins in the uterine tissue of intrauterine adhesion mice. C group, Control group; M group, laparotomy was used to construct a model of intrauterine adhesion; NT group was treated with *L. crispatus* for intrauterine adhesion mice; ET group was treated with *L. crispatus*-pMG36emCXCL12 for intrauterine adhesion mice. ns, P > 0.05; *P < 0.05; **P < 0.01; ***P < 0.001. Two animal models (IUA and IUA with diabetes) were established and analyzed concurrently within the same experimental batch and that the same Group C control samples from the same experimental batch were intentionally shared across both models.

In our previous work, we constructed engineered commensal bacteria that expressed mCXCL12, and the expression level was about 94 pg/mL determined by ELISA (**Figure 1A**). In addition, to increase the reliability of the engineered commensal bacteria, we carried out the stability test of the recombinant strain. The results showed that 100% of the plasmids were still present on day 7 after culture, while 83% of the plasmids were still present in the engineered symbiotic bacteria after 1 month of culture (**Table S2**). As shown in **Figure 1B**, during the development and administration of the model of intrauterine adhesion mice, the control group (C, n = 2), the intrauterine adhesion mice group (M, n = 3), the *L. crispatus*-treated intrauterine adhesion mice group (NT, n = 3), and the *L. crispatus*-pMG36e-mCXCL12-treated intrauterine adhesion mice group (ET, n = 2) respectively died of model development-related factors within 24 hours. Finally, eight mice survived in the C and ET groups, seven in the M group and NT groups. After seven consecutive administrations, the isolated uterus of mice in group M lost elasticity and the surface of the uterus was pale and dried out compared to group C. In contrast, the uterus of the NT group and ET group still had some elasticity and the uterine shape was maintained significantly better than that of the group M. In **Figure 1C**, H&E and Masson staining further showed that the uterine tissue in the M group was severely congested, with reduced endometrial glandular and collagen staining with inflammatory cell infiltration, and the integrity of the endometrium was disrupted. After the intervention of *L. crispatus* and *L. crispatus*-pMG36e-mCXCL12, the number of endometrial glands and collagen staining increased and the inflammatory response was significantly improved. This indicates that *L. crispatus* and *L. crispatus*-pMG36e-mCXCL12 reduced the infiltration of inflammatory cells, the formation of collagen fibres and the destruction of the endometrium compared to group M.

The TGF-β1/Smads signalling pathway plays an important regulatory role in the pathophysiology of fibrosis in various organs. Therefore, we evaluated their expression levels in the uterus of each group. As shown in **Figures 1D, F–I**, surgery resulted in a significant increase in the expression levels of TGF-β1 (1.00 vs. 4.85, P> 0.05), p-Smad2 (1.00 vs. 3.05, P < 0.05) and p-Smad3 (1.00 vs. 2.40, P < 0.05) compared with the control group, whereas treatment with *L. crispatus* and *L. crispatus*-pMG36e-mCXCL12 reversed this trend, restoring the expression levels of TGF-β1, p−Smad2 and p−Smad3 in the NT group to 4.18, 2.61 and 1.56 and in the ET group to 3.62, 1.38 and 0.98, respectively. In addition, the therapeutic effect of *L. crispatus*-pMG36e-mCXCL12 was greater than that of *L. crispatus*, with the former significantly reducing the expression levels of p-Smad2 and p-Smad3. We further investigated the expression of the fibrotic degradation factor MMP9, which was markedly inhibited by surgery (1.00 vs. 0.29, P < 0.01) and significantly increased by probiotic treatment (0.29 vs. 0.65 vs. 0.97), while the effect of *L. crispatus*-pMG36e-mCXCL12 treatment was more pronounced (P < 0.01).

**Figure 2:**
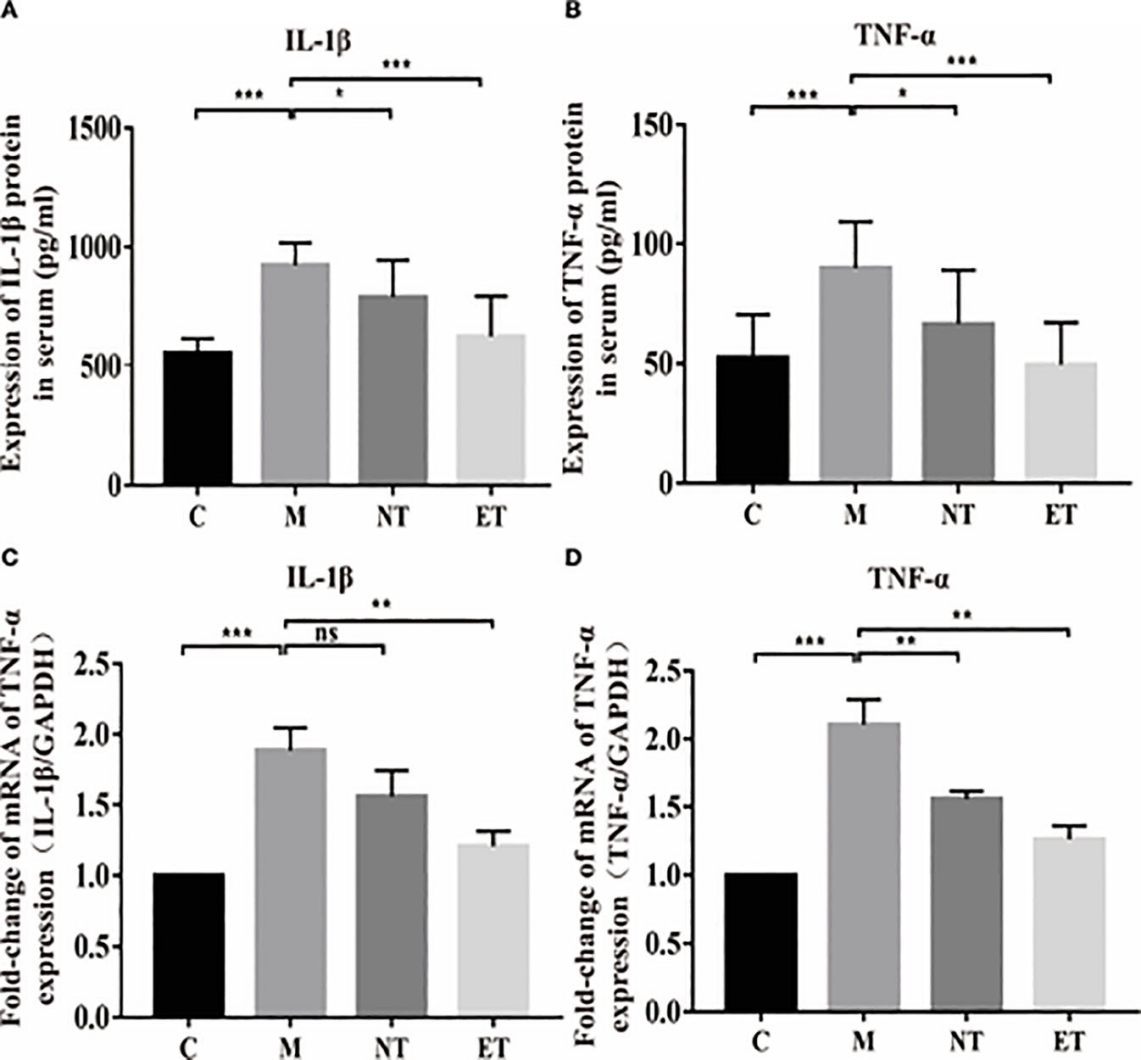
Effects of *L. crispatus*-pMG36e-mCXCL12 on the expression of pro-inflammatory factors at the protein and gene levels in intrauterine adhesion mice. Effect of *L. crispatus*-pMG36e-mCXCL12 on the expression of IL-1β **(A)** and TNF-α **(B)** in the uterine tissue of intrauterine adhesion mice at the protein level. Effect of *L. crispatus*-pMG36e-mCXCL12 on the expression of IL-1β **(C)** and TNF-α **(D)** in the uterine tissue of intrauterine adhesion mice at the gene level. C group, Control group; M group, laparotomy was used to construct a model of intrauterine adhesion; NT group was treated with *L. crispatus* for intrauterine adhesion mice; ET group was treated with *L. crispatus*-pMG36e-mCXCL12 for intrauterine adhesion mice. ns, P > 0.05; *P < 0.05; **P < 0.01; ***P < 0.001. Two animal models (IUA and IUA with diabetes) were established and analyzed concurrently within the same experimental batch and that the same Group C control samples from the same experimental batch were intentionally shared across both models.

To explore whether the occurrence of IUA is related to inflammation, we further investigated the classical inflammatory TLR4/NF-κB signalling pathway using western blotting. As shown in **Figures 1E, J, K**, surgery increased TLR4 (1.00 vs. 2.22, P < 0.01) and p-NF-κB/NF-κB (1.00 vs. 4.52, P < 0.001) compared with the control group, whereas treatment with *L. crispatus* and *L. crispatus*-pMG36e-mCXCL12 respectively, reduced the expression levels of TLR4 (2.22 vs. 1.62 vs. 1.13) and p-NF-κB (4.52 vs. 3.25 vs. 1.15) in the groups NT and ET.

### *crispatus*-pMG36e-mCXCL12 Decreased the Production of Pro-Inflammatory Factors in the Intrauterine Adhesion Mice

Activation of the TLR4/NF-κB signalling pathway will lead to the release of pro-inflammatory factors. Therefore, we further investigated the effects of *L. crispatus* and *L. crispatus*-pMG36e-mCXCL12 on pro-inflammatory factors at protein (ELISA) and gene (q-PCR) levels. As shown in **Figures 2A–D**, surgery significantly increased the expression of pro-inflammatory factors IL-1β (553.80 vs. 922.50, P < 0.001) and TNF-α (52.44 vs. 89.97, P < 0.001) in serum, and treatment with probiotics significantly reduced the expression of the pro-inflammatory factors in group ET. Similarly, q-PCR results supported this finding that surgery boosted the transcriptional levels of IL-1β (1.00 vs. 1.88, P < 0.001) and TNF-α (1.00 vs. 2.10, P < 0.001) in the group M compared with the group C, and treatment with *L. crispatus* and *L. crispatus*-pMG36e-mCXCL12 reduced the expression of the pro-inflammatory factors IL-1β and TNF-α in the group ET.

### Effects of *L. crispatus*-pMG36e-mCXCL12 on the Vaginal Microbiota in the Intrauterine Adhesion Mice

**Figure 3:**
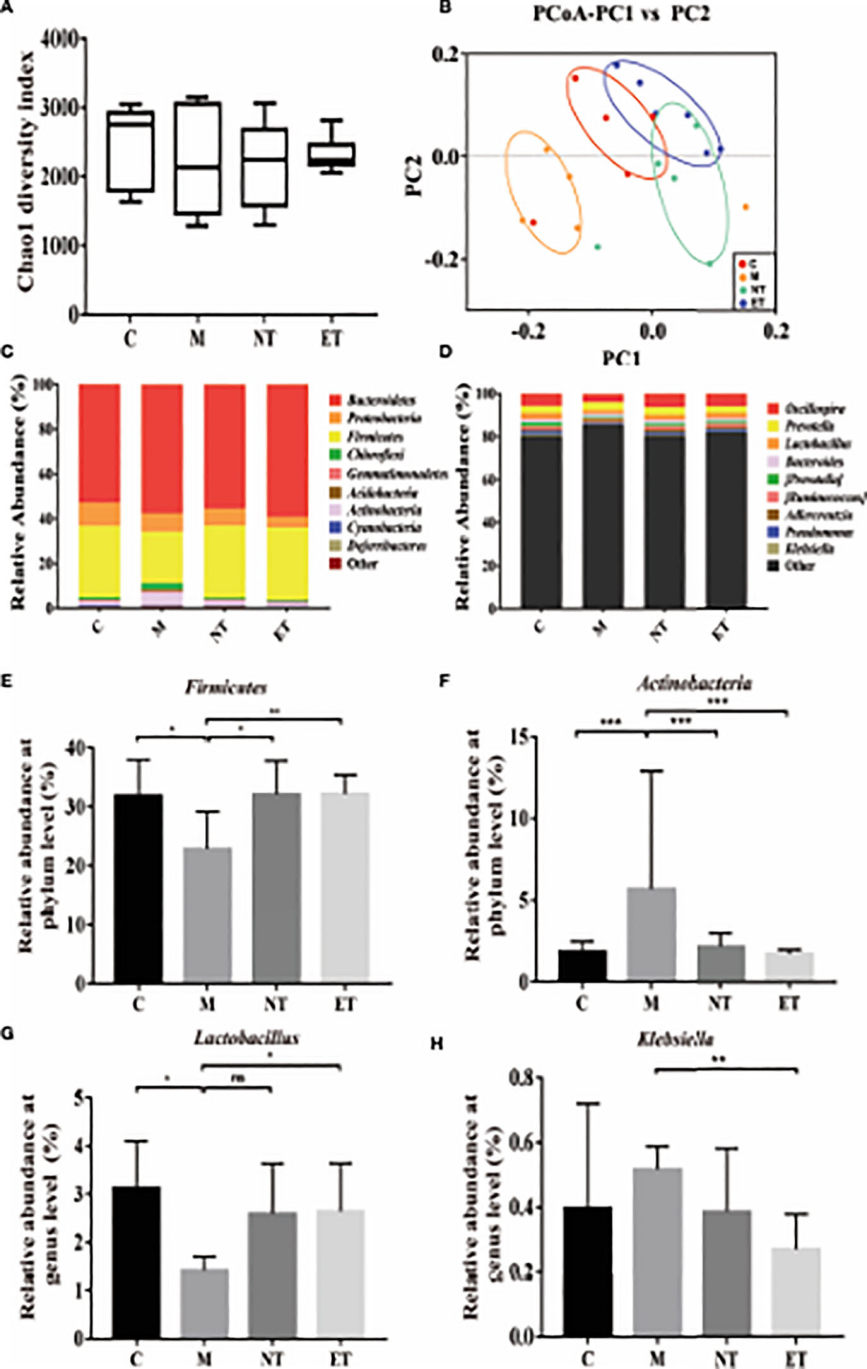
Effects of *L. crispatus*-pMG36e-mCXCL12 on vaginal microbiota in intrauterine adhesion mice. Evaluation of the effect of *L. crispatus*-pMG36emCXCL12 on vaginal microbiota of intrauterine adhesion mice using **(A)** the Chao1 Diversity, **(B)** the PCoA of the β diversity index, the relative abundance **(C)** at the phylum level and **(D)** at the genus level. Evaluation of the effect of *L. crispatus*-pMG36e-mCXCL12 on the relative abundances of the phyla **(E)** *Firmicutes*, **(F)** *Actinobacteria* in intrauterine adhesion mice; Evaluation of the effect of *L. crispatus*-pMG36e-mCXCL12 on the relative abundances of the genera **(G)** *Lactobacillus*, **(H)** *Klebsiella* in intrauterine adhesion mice. C group, Control group; M group, laparotomy was used to construct a model of intrauterine adhesion; NT group was treated with *L. crispatus* for intrauterine adhesion mice; ET group was treated with *L. crispatus*-pMG36e-mCXCL12 for intrauterine adhesion mice. ns, P > 0.05; *P < 0.05; **P < 0.01; ***P < 0.001. Two animal models (IUA and IUA with diabetes) were established and analyzed concurrently within the same experimental batch and that the same Group C control samples from the same experimental batch were intentionally shared across both models.

In our previous research, we confirmed that the vaginal microbiota was closely associated with IUA using high-throughput sequencing (26). In the present study, we compared the vaginal microbiota of normal mice, intrauterine adhesion mice, and probiotic-treated mice using high-throughput sequencing. As shown in **Figure 3**, a higher Chao1 index (**Figure 3A**) was obtained for group M compared with group C, and treatment with *L. crispatus* and *L. crispatus*-pMG36e-mCXCL12 in groups NT and ET, respectively, restored bacterial homeostasis. In **Figure 3B**, the PCoA results indicate that most of the dots in the C, NT and ET groups were far from those in the M group. Subsequently, we investigated the top 10 microbial taxa in the vaginal microbiota of normal mice, intrauterine adhesion mice and probiotic-treated mice at the phylum level (**Figure 3C**) and the genus level (**Figure 3D**). At the phylum level, the communities of the vaginal microbiota in group M were characterized by a decrease in *Firmicutes* (31.88 vs. 22.81%, P < 0.05) and an increase in *Actinobacteria* (1.84 vs. 5.66%, P < 0.001) compared to the group C. However, supplementation with *L. crispatus*-pMG36e-mCXCL12 altered the relative abundance of *Firmicutes* (22.81 vs. 32.09 vs. 32.2%) and *Actinobacteria* (5.66 vs. 2.14 vs. 1.68%) in groups NT and ET (**Figures 3E, F**). At the genus level, the vaginal microbiota of group M showed a lower abundance of probiotics and *Lactobacillus* (3.13 vs. 1.42%; P < 0.05) and a higher abundance of the pathogen *Klebsiella* (0.40 vs. 0.52%) compared to the group C. Supplementation with *L. crispatus*-pMG36e-mCXCL12 reversed this trend, with the NT and ET groups exhibiting similar abundances of *Lactobacillus* (2.59 vs. 2.64%) and *Klebsiella* (0.39 vs. 0.27%) compared to the control group (**Figures 3G, H**).

### *crispatus*-pMG36e-mCXCL12 Prevented Intrauterine Adhesions After Intrauterine Surgery in Diabetic Mice by Inhibiting the Formation of Fibrosis and Inflammation

**Figure 4:**
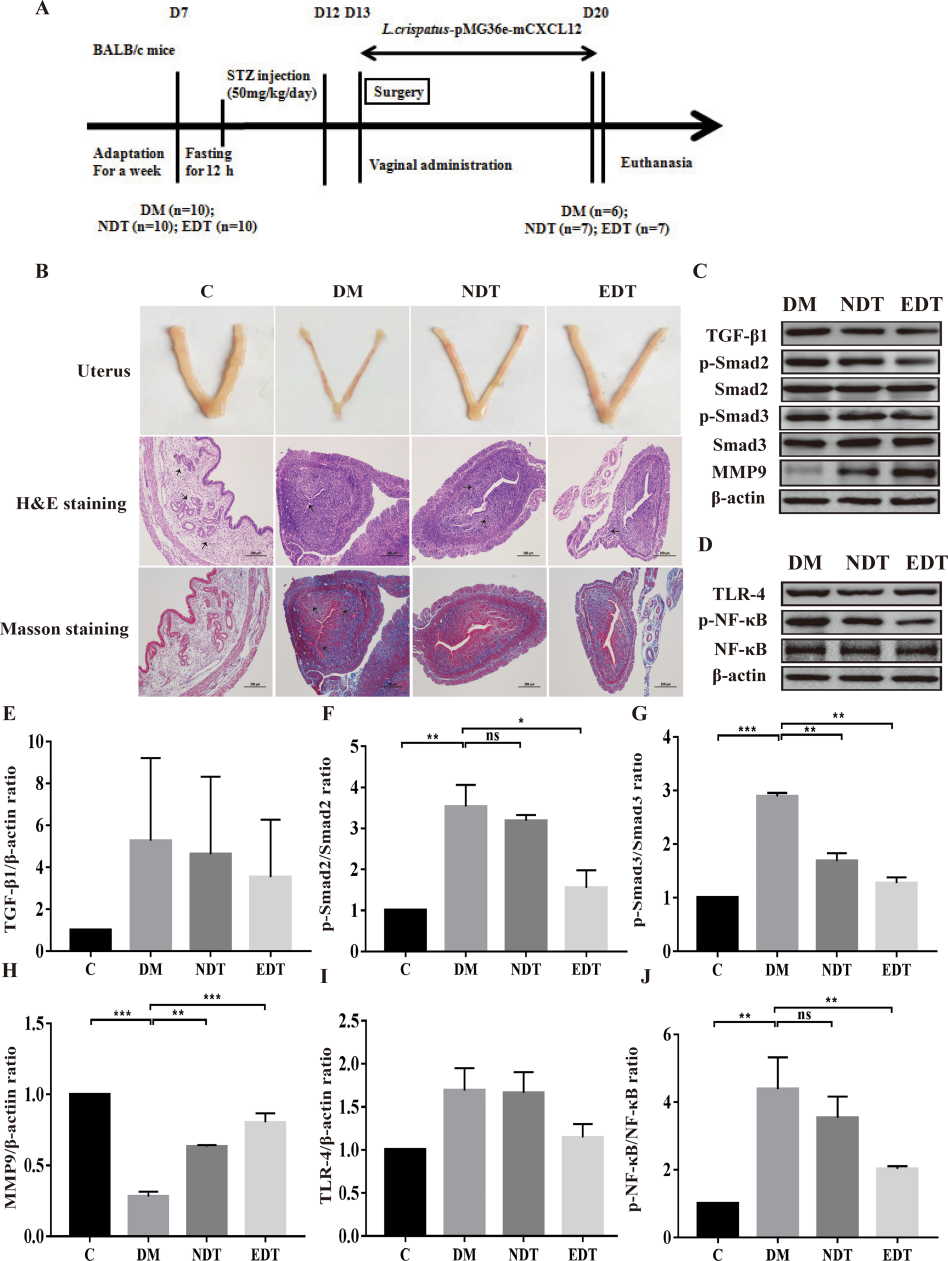
Effect of *L. crispatus*-pMG36e-mCXCL12 on the expression of proteins associated with the TGF- β1/Smads and TLR4/NF-κB signalling pathway in intrauterine adhesion mice with diabetes. **(A)** Experimental scheme to evaluate the effects of *L. crispatus*-pMG36e-mCXCL12 on the prevention of intrauterine adhesion in diabetic mice. **(B)** H&E staining was used to observe uterine inflammation, and Masson staining was used to observe collagen deposition in the uterine tissue of intrauterine adhesion mice with diabetes (magnification: × 100). **(C)** Expression of fibrotic-related proteins in the uterine tissue of intrauterine adhesion mice with diabetes. **(D)** Expression of inflammatory-related proteins in the uterine tissue of intrauterine adhesion mice with diabetes. Effect of *L. crispatus*-pMG36e-mCXCL12 on fibrotic-related TGF-β1 **(E)**, p-Smad2/Smad2 **(F)**, p-Smad3/Smad3 **(G)**, MMP-9 **(H)** proteins in the uterine tissue of intrauterine adhesion mice with diabetes. Effect of L. crispatus-pMG36e-mCXCL12 on inflammatory-related TLR4 **(I)**, p-NF-κB/NF-κB **(J)** proteins in the uterine tissue of intrauterine adhesion mice with diabetes. C group, Control group; DM group, laparotomy was used to construct a model of intrauterine adhesion mice with diabetes; NDT group was treated with *L. crispatus* for intrauterine adhesion mice with diabetes; EDT group was treated with L. crispatus-pMG36e-mCXCL12 for intrauterine adhesion mice with diabetes. ns, P > 0.05; *P < 0.05; **P < 0.01; ***P < 0.001. Two animal models (IUA and IUA with diabetes) were established and analyzed concurrently within the same experimental batch and that the same Group C control samples from the same experimental batch were intentionally shared across both models.

Meanwhile, we evaluated the formation of fibrosis and inflammation of uterus in the intrauterine adhesion mice with diabetes. As shown in **Figures 4A, B**, during the development and administration of the model of intrauterine adhesion and diabetes mice, the intrauterine adhesion mice with diabetes (DM, n = 2), *L. crispatus*-treated intrauterine adhesion mice with diabetes (NDT, n = 3) and *L. crispatus*-pMG36e-mCXCL12-treated intrauterine adhesion mice with diabetes (EDT, n = 4) respectively died of model developing-related factors within 24 hours. Finally, six mice survived in the DM group and seven in the M group and NT groups. In addition, similar to the intrauterine adhesion mice, the pale uterine atrophy and loss of elasticity were observed in the intrauterine adhesion mice with diabetes (DM group). The morphology of the uterus was significantly improved with the intervention of *L. crispatus* and *L. crispatus*-pMG36e-mCXCL12.

To further explore the effects of *L. crispatus* and *L. crispatus*-pMG36e-mCXCL12 on the formation of fibrosis and inflammation in the intrauterine adhesions mice with diabetes, we compared the TGF-β1 and p-Smad2 levels and found that, as shown in **Figures 4C, E–G**, surgery resulted in the expression levels of TGF-β1 (1.00 vs. 5.27, P > 0.05), p-Smad2 (1.00 vs. 3.53, P < 0.05) and p−Smad3 (1.00 vs. 2.89, P < 0.05) showed an increasing trend compared with the control group, whereas treatment with *L. crispatus* and *L. crispatus*-pMG36e-mCXCL12 reversed this trend and restored the expression levels of TGF-β1, p−Smad2 and p−Smad3 in the NDT group to 4.62, 3.18 and 1.69 and in the EDT group to 3.52, 1.55 and 1.27, respectively. *L. crispatus*-pMG36e-mCXCL12 treatment significant reduced the expression levels of p−Smad2 and p-Smad3, suggesting the therapeutic effect of *L. crispatus*-pMG36e-mCXCL12 was more obvious than that of *L. crispatus*, and the former significantly reduced the expression levels of p−Smad2 and p-Smad3. We further investigated the expression of the fibrotic degradation factor MMP9, which was markedly inhibited by surgery (1.00 vs. 0.28, P < 0.05), while probiotic treatment significantly increased MMP9 expression (0.28 vs. 0.63 vs. 0.80), and the effect of *L. crispatus*-pMG36e-mCXCL12 treatment was more pronounced (**Figure 4H**).

In addition, we also explored the correlation between the development of intrauterine adhesions and inflammation in diabetic mice. As shown in **Figures 4D, I, J**, surgery increased TLR4 (1.00 vs 1.69, P > 0.05) and p-NF-κB/NF-κB (1.00 vs 4.39, P < 0.01) compared with controls, while treatment with *L. crispatus* and *L. crispatus*-pMG36e-mCXCL12, respectively, decreased the NDT group and EDT.

### *crispatus*-pMG36e-mCXCL12 Decreased the Production of Pro-Inflammatory Factors in the Intrauterine Adhesion Mice With Diabetes

**Figure 5:**
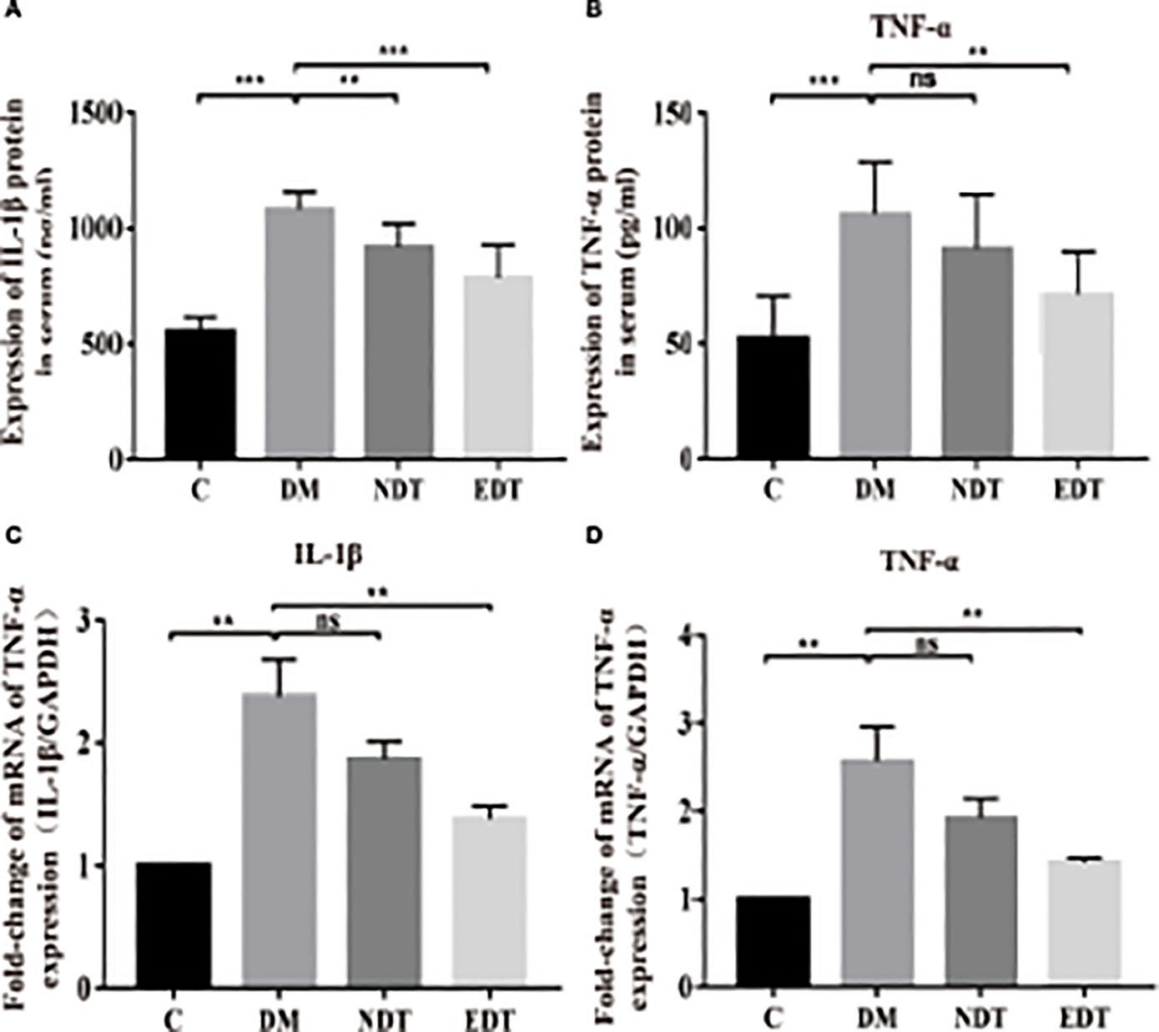
Effects of *L. crispatus*-pMG36e-mCXCL12 on the expression of pro-inflammatory factors at the protein and gene levels in intrauterine adhesion mice with diabetes. Effect of *L. crispatus*-pMG36e-mCXCL12 on the expression of IL-1β (A) and TNF-α (B) in the uterine tissue of intrauterine adhesion mice with diabetes at the protein level. Effect of *L. crispatus*-pMG36e-mCXCL12 on the expression of IL-1β (C) and TNF-α (D) in the uterine tissue of intrauterine adhesion mice with diabetes at the gene level. C group, Control group; DM group, laparotomy was used to construct a model of intrauterine adhesion mice with diabetes; NDT group was treated with *L. crispatus* for intrauterine adhesion mice with diabetes; EDT group was treated with *L. crispatus*-pMG36e-mCXCL12 for intrauterine adhesion mice with diabetes. ns, P > 0.05; *P < 0.05; **P < 0.01; ***P < 0.001. Two animal models (IUA and IUA with diabetes) were established and analyzed concurrently within the same experimental batch and that the same Group C control samples from the same experimental batch were intentionally shared across both models.

In the experimental group of intrauterine adhesion mice with diabetes, we further investigated the effects of *L. crispatus* and *L. crispatus*-pMG36e-mCXCL12 on pro-inflammatory factors at protein (ELISA) and gene (q-PCR) levels and obtained a similar result. As shown in **Figures 5A–D**, surgery significantly increased the expression of pro-inflammatory factors IL-1β (553.80 vs. 1080.00, P < 0.001) and TNF-α (52.44 vs. 106.00, P < 0.001) in the serum, and treatment with probiotics significantly reduced the expression of the pro-inflammatory factors in groups EDT. Likewise, the q-PCR results supported this finding that surgery upregulated the transcriptional levels of IL-1β (1.00 vs. 2.37, P < 0.01) and TNF-α (1.00 vs. 2.55, P < 0.01) in group DM compared to group C, and treatment with *L. crispatus*-pMG36e-mCXCL12 significantly reduced the expression of the pro-inflammatory factors IL-1β and TNF-α in groups EDT.

### Effects of *L. crispatus*-pMG36e-mCXCL12 on the Vaginal Microbiota in the Intrauterine Adhesion Mice With Diabetes

**Figure 6:**
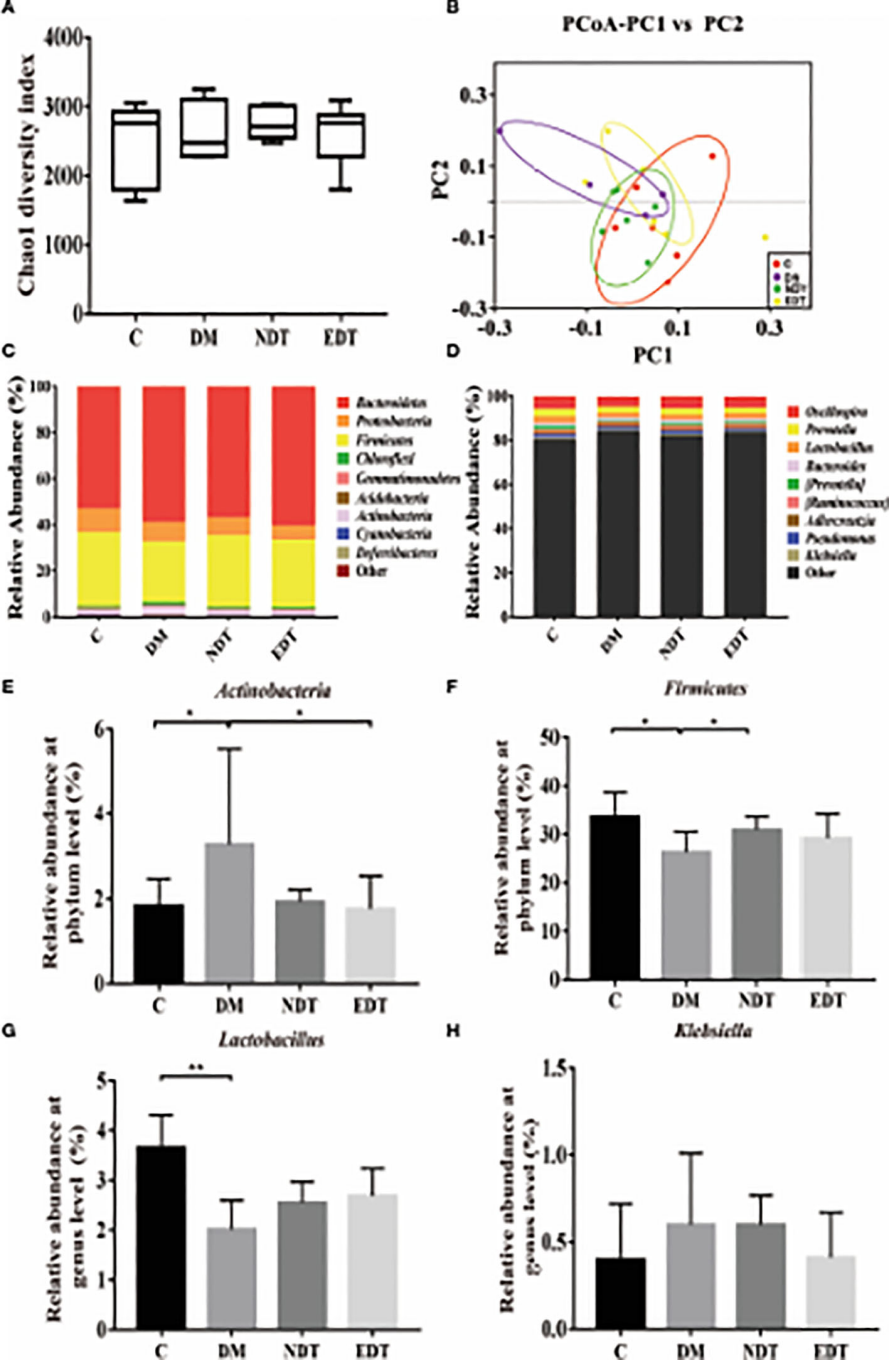
Effects of *L. crispatus*-pMG36e-mCXCL12 on vaginal microbiota in intrauterine adhesion mice with diabetes. Evaluation of the effect of *L. crispatus*-pMG36e-mCXCL12 on vaginal microbiota of intrauterine adhesion mice with diabetes using **(A)** the Chao1 Diversity, **(B)** the PCoA of the β diversity index, the relative abundance **(C)** at the phylum level and **(D)** at the genus level. Evaluation of the effect of *L. crispatus*-pMG36e-mCXCL12 on the relative abundances of the phyla **(E)** *Firmicutes*, **(F)** *Actinobacteria* in intrauterine adhesion mice with diabetes; Evaluation of the effect of *L. crispatus*pMG36e-mCXCL12 on the relative abundances of the genera **(G)** *Lactobacillus*, **(H)** *Klebsiella* in intrauterine adhesion mice with diabetes. C group, Control group; DM group, laparotomy was used to construct a model of intrauterine adhesion mice with diabetes; NDT group was treated with *L. crispatus* for intrauterine adhesion mice with diabetes; EDT group was treated with *L. crispatus*-pMG36e-mCXCL12 for intrauterine adhesion mice with diabetes. ns, P > 0.05; *P < 0.05; **P < 0.01; ***P < 0.001. Two animal models (IUA and IUA with diabetes) were established and analyzed concurrently within the same experimental batch and that the same Group C control samples from the same experimental batch were intentionally shared across both models.

Similarly, we further compared the vaginal microbiota of normal mice, intrauterine adhesion mice with diabetes, and probiotic-treated mice using high-throughput sequencing. As shown in **Figure 6A**, the DM group obtained a higher Chao1 index compared to the C group, and treatment with *L.crispatus* and *L.crispatus*-pMG36e-mCXCL12 restored bacterial homeostasis in the NDT and EDT groups, respectively. In **Figure 6B**, the PCoA results indicated that most of the dots in the C, NDT and EDT groups were dispersed with those in the DM group. Then we analysed the top 10 microbial taxa in the vaginal microbiota of normal mice, intrauterine adhesion mice with diabetes, and probiotic-treated mice at the phylum level (**Figure 6C**) and the genus level (**Figure 6D**). In the experimental group of intrauterine adhesion mice with diabetes, we obtained similar results, supplementation with *L. crispatus*-pMG36e-mCXCL12 significantly changed the composition of the vaginal microbial community, with a greatly enhanced richness of *Firmicutes* (for C, DM, NDT and EDT, the levels were 33.59, 26.21, 30.85 and 29.19%, respectively) and a diminished richness of *Actinobacteria* (1.84, 3.28, 1.92 and 1.76%, respectively) (**Figures 6E, F**). At the genus level, surgery markedly reduced the relative abundances of beneficial bacteria such as *Lactobacillus* (3.66 vs. 2.01%, P < 0.05), but greatly increased the relative abundance of the pathogenic genus *Klebsiella* (0.40 vs. 0.60%). In contrast, *L.crispatus*-pMG36e-mCXCL12 treatment in groups NDT and EDT greatly increased the abundance of *Lactobacillus* (2.01 vs. 2.55 vs. 2.68%), but decreased *Klebsiella* (0.60 vs. 0.598%vs. 0.41%) (**Figures 6G, H**).

## Discussion

In recent years, IUA has become one of the most common causes of abnormal menstruation and reproductive dysfunction in women of childbearing age (27). There are relevant studies on how to reduce the recurrence of re-adhesion in patients with IUA, such as intrauterine devices, balloons, uterine cavity anti-adhesives and amniotic membranes placed in the uterine cavity to prevent re-adhesion (28), but the effect is not satisfactory. Therefore, how effectively reducing the formation of postoperative adhesions in IUA patients is an urgent problem for gynaecologists. Vågesjö et al. developed an engineered commensal bacterium to express CXCL12 and found that CXCL12-delivering bacteria administrated locally to mouse wounds efficiently enhanced wound closure and also improved wound closure in diabetic mice (29). Inspired by Vågesjö’s work, we transferred the DNA sequence encoding CXCL12 into *Lactobacillus crispatus* using pMG36e as a vector, which allowed the cells to produce lactic acid and also continuously secrete CXCL12. In parallel, we developed two animal models of intrauterine adhesion mice without diabetes and intrauterine adhesion mice with diabetes, and found that *L. crispatus*-pMG36e-mCXCL12 significantly improved uterine inflammation and fibrosis in model mice.

Intrauterine operation is the main factor leading to IUA, which is frequently characterised by damage of the basal layer of the endometrium, endometrial fibrosis caused by continuous inflammation and blocked regeneration and reparation (30). According to previous studies, TGF-β1 increases the synthesis and expression of collagen, glycoproteins, etc., while decreasing the synthesis of matrix-degrading proteases in the maintenance of extracellular matrix homeostasis (31). Studies have indicated that TGF-β1 can activate Smad2 and Smad3, which then activate myofibroblasts, leading to excessive production of extracellular matrix (ECM) and inhibition of ECM degradation, thereby inducing renal fibrosis and ultimately chronic renal failure (32). In contrast, the physiological role of MMP9 is opposite to that of TGF-β1, it is a protease that breaks down the extracellular matrix. Upon activation by extracellular protease cleavage, it degrades extracellular matrix components such as collagen IV and V, thereby reducing the accumulation of extracellular matrix (33). In the present study, we found that surgery increased fibrosis both in intrauterine adhesion mice and intrauterine adhesion mice with diabetes, and *L. crispatus*-pMG36e-mCXCL12 can significantly reduce the key proteins related to the TGF-β1/Smads pathway (TGF-β1, p-Smad2, p-Smad3), increasing the level of fibrosis-inhibiting protein MMP9, suggesting that this technique can prevent the development of intrauterine adhesions by inhibiting the fibrotic response.

In addition to uterine cavity surgical trauma, infection is also an important cause of IUA. The deposition of extracellular matrix produced in the inflammatory response can promote tissue repair, but excessive deposition can cause tissue fibrosis diseases, ultimately leading to a series of pathological and physiological changes (34). It has been shown that TLR4 expression is up-regulated after trauma or tubal interstitial inflammation, which is associated with wound healing and cicatricial stenosis of the fallopian tube (35). TLR4 is an important molecule that mediates the body’s natural immune response and plays an important role in non-infectious tissue damage and tissue repair (36). Ligand-activated TLR4 activates the transcription of NF-kB through the MyD88-dependent signal transduction pathway, and NF-kB is a protein that has transcriptional activation function for many inflammatory mediators (37), mediating the transcriptions of pro-inflammatory factors such as IL-1β and TNF-α. These pro-inflammatory factors act on fibroblasts and ultimately lead to the formation of tissue fibrosis (38). Therefore, we further evaluated the effect of probiotics on the inflammatory status of mice and found that *Lactobacillus crispatus* can significantly down-regulate the TLR4/NF-κB inflammatory signalling pathway, reducing the levels of pro-inflammatory factors IL-1β and TNF-α, and *L. crispatus*-pMG36e-mCXCL12 can ameliorate inflammation even more significantly.

There is increasing evidence that the vaginal microbiota plays an important role in maintaining the stability of the vaginal environment (39). Disturbance of the microbiota not only cause local inflammation, but the pathogenic bacteria can spread to the uterine cavity and increase the risk of uterine cavity infection (40). To evaluate the effect of *L. crispatus*-pMG36e-mCXCL12 on the vaginal microbiota, we sequenced the 16S rRNA gene to detect the presence of vaginal microorganisms in mice. High-throughput sequencing results revealed that probiotics greatly increased the abundance of *Lactobacillus* spp. while reducing the the number of pathogenic *Klebsiella* spp. *Lactobacilli* are the most common bacteria in the vagina of healthy women. The ability of *Lactobacillus* to adhere to and compete for vaginal epithelial adhesion sites and produce antimicrobial compounds (hydrogen peroxide, lactic acid, bacteriocin-like substances) is of great importance for the prevention of colonisation by pathogens (41). A recent study by Ettore Palma et al. confirmed that long-term supplementation with *Lactobacilli* in women with HPV infection complicated by bacterial vaginosis or vaginitis could help resolve viral infections by re-establishing the original eubiosis (42). *Klebsiella* is an important conditional pathogenic bacterium in the respiratory tract and the intestines of humans. It can cause respiratory infections, urinary system infections, wound infections and diarrhoea and is even associated with severe sepsis, meningitis and peritonitis. Studies have shown that some *Klebsiella* strains can produce toxic factors such as lipopolysaccharides, which further activate TLR4 to induce fibrosis and inflammation (43). In addition, Yun et al. have shown that LPS-induced endometrial infection is closely associated with the development of IUA, suggesting that *Klebsiella* may be involved in the development of IUA (44).

As we all know, the incidence of poor wound healing is higher in diabetic patients than in healthy individuals, but it remains controversial whether hyperglycemia has an effect on the incidence of uterine adhesions after intrauterine surgery (45). Therefore, we further evaluated the effect of *L. crispatus* and *L. crispatus*-pMG36e-mCXCL12 on the formation of intrauterine adhesions in diabetic mice and obtained similar results to non-diabetic mice. We found that *L.crispatus* and *L.crispatus*-pMG36e-mCXCL12 significantly ameliorated fibrosis and inflammation in the uterine cavity of diabetic mice, and restored vaginal microbiota balance in diabetic mice. The main reason may be due to the complex mechanism of poor wound healing in diabetes, abnormal inflammatory response, decreased granulation tissue content, peripheral neuropathy and impaired angiogenesis at the wound are all involved in inducing wound healing disorders (46). Current researches have demonstrated that a variety of molecules can exert their biological effects by directly acting on wound tissue. The imbalance of these molecules will directly hinder inflammation regression, angiogenesis, granulation tissue formation and other links, resulting in poor wound healing (47). The up-regulation of TGFβ/SMADs signaling pathway will exacerbate fibrosis, the low expression of MMP will lead to the degradation of the extracellular matrix and exacerbate fibrosis, and disruption of inflammatory factors will enhance and prolong the inflammatory response of wounds (48). However, *L.crispatus*-pMG36e-mCXCL12 alleviated the fibrosis and inflammatory response of the uterine cavity of diabetic mice and prevented the occurrence of IUA by restoring the balance of vaginal microbiota while continuously delivering CXCL12.

In summary, our results lead us to conclude that administering *L. crispatus* and *L. crispatus*-pMG36e-mCXCL12 to the model mice alleviated IUA by restoring the microbial diversity and reducing endometrial fibrosis and inflammation. Therefore, our findings suggested that vaginal administration of probiotics has a positive effect on preventing IUA after intrauterine surgery. However, the mouse model of intrauterine adhesions cannot fully mimic the actual situation in humans, and we detected no CXCL4+ immune cells. In this sense, further studies are needed to advance the potential clinical application of an engineered strain that carries CXCL12 for human disease treatment.

## Data Availability Statement

The datasets presented in this study can be found in online repositories. The names of the repository/repositories and accession number(s) can be found in the article/**Supplementary Material**.

## Ethics Statement

The animal study was reviewed and approved by Laboratory Animal Ethics Committee of Nanchang Royo Biotech Co,. Ltd.

## Author Contributions

TC, ZL, and XD designed the study. YK, QX, FW, and LH carried out the experiments. All authors read and approved the manuscript and agree to be accountable for all aspects of the research in order to ensure that the integrity of any part of the study.

## Funding

This study was supported by grants from the National Natural Science Foundation of China (grant no. 81960103, 82060638), the Natural Science Foundation of JiangXi province (grant no. 20202BABL206021), the Science and Technology Research Project of JiangXi Education Department (grant no. GJJ180013) and the Double Thousand Plan of Jiangxi Province (High-End Talents Project of scientific and technological innovation).

## Conflict of Interest

The authors declare that the research was conducted in the absence of any commercial or financial relationships that could be construed as a potential conflict of interest.

## Publisher’s Note

All claims expressed in this article are solely those of the authors and do not necessarily represent those of their affiliated organizations, or those of the publisher, the editors and the reviewers. Any product that may be evaluated in this article, or claim that may be made by its manufacturer, is not guaranteed or endorsed by the publisher.
