# Correction: Protective effects of engineered *Lactobacillus crispatus* on intrauterine adhesions in mice *via* delivering CXCL12

**DOI:** 10.3389/fimmu.2026.1794467

**Published:** 2026-03-24

**Authors:** Yao Kong, Zhaoxia Liu, Qin Xiao, Fei Wu, Lijuan Hu, Xiaorong Deng, Tingtao Chen

**Affiliations:** 1 Department of Obstetrics and Gynecology, The Second Affiliated Hospital of Nanchang University, Nanchang, China; 2 Department of Gastrointestinal Surgery, The Second Affiliated Hospital of Nanchang University, Nanchang, China; 3 National Engineering Research Center for Bioengineering Drugs and The Technologies, Institute of Translational Medicine, Nanchang University, Nanchang, China

**Keywords:** *Lactobacillus crispatus*, mCXCL12, pMG36e, intrauterine adhesions, high-throughput sequencing

There was a mistake in **Figure 4** as published. We have identified unintentional misuse of images for the Western blot strips p-Smad3 and p-NF-κB in **Figures 4C, D**. We confirmed that the problem was due to misplacement during image integration. We have now re-examined the original dataset and replaced the affected **Figure 4C** with the correct and unused image from the same experimental batch. The corrected **Figure 1** appears below.

**Figure 1:**
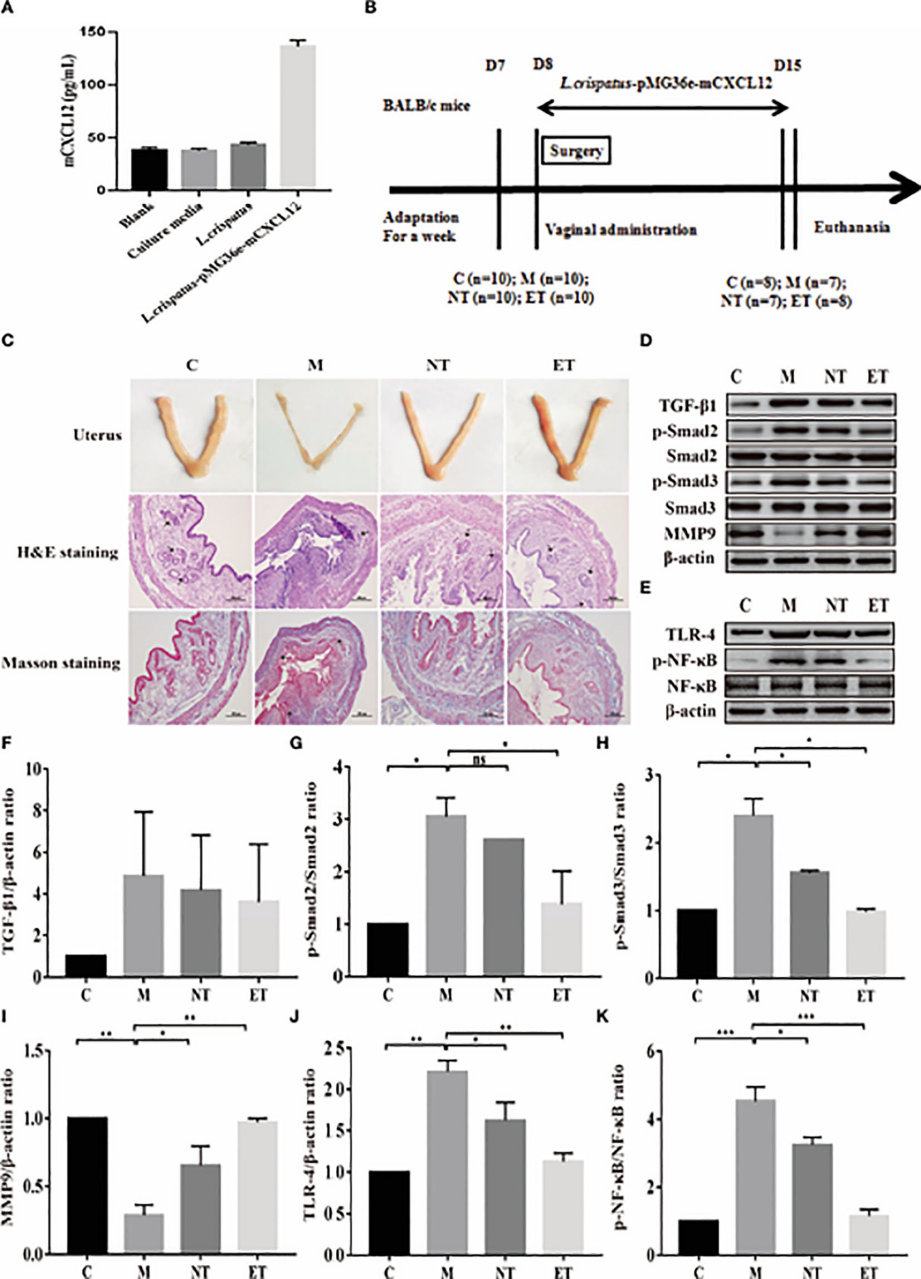
Effect of *L. crispatus*-pMG36e-mCXCL12 on the expression of proteins associated with the TGF-β1/Smads and TLR4/NF-κB signalling pathway in intrauterine adhesion mice. **(A)** mCXCL12 *in-vitro* bacterial expression. **(B)** Experimental scheme to evaluate the effects of *L. crispatus*-pMG36e-mCXCL12 on the prevention of intrauterine adhesion in mice. **(C)** H&E staining was used to observe uterine inflammation, and Masson staining was used to observe collagen deposition in the uterine tissue of intrauterine adhesion mice (magnification: × 100). **(D)** Expression of fibrotic-related proteins in the uterine tissue of intrauterine adhesion mice. **(E)** Expression of inflammatory-related proteins in the uterine tissue of intrauterine adhesion mice. Effect of *L. crispatus*-pMG36e-mCXCL12 on fibrotic-related TGF-β1 **(F)**, p-Smad2/Smad2 **(G)**, p-Smad3/Smad3 **(H)**, MMP-9 **(I)** proteins in the uterine tissue of intrauterine adhesion mice. Effect of *L. crispatus*-pMG36e-mCXCL12 on inflammatory-related TLR4 **(J)**, p-NF-κB/NF-κB **(K)** proteins in the uterine tissue of intrauterine adhesion mice. C group, Control group; M group, laparotomy was used to construct a model of intrauterine adhesion; NT group was treated with *L. crispatus* for intrauterine adhesion mice; ET group was treated with *L. crispatus*-pMG36e-mCXCL12 for intrauterine adhesion mice. ns, P > 0.05; *P < 0.05; **P < 0.01; ***P < 0.001. Two animal models (IUA and IUA with diabetes) were established and analyzed concurrently within the same experimental batch and that the same Group C control samples from the same experimental batch were intentionally shared across both models.

There was a mistake in the caption of **Figures 1**–**6** as published. In the original study design, We established two animal models: intrauterine adhesion (IUA) mice and IUA mice with diabetes. And all experiments were performed concurrently within the same experimental batch. During the original peer-review process, based on the suggestions of the handling editor and reviewers, we reorganized the Results section by separating the two animal models to improve clarity and logical structure. However, because the experiments were conducted simultaneously, the same Group C control samples were used for comparative analyses across both model presentations. This shared-control design was discussed and explained during the peer-review process and was acknowledged by the editor and reviewers at that time. However, this information was not explicitly disclosed in the final published figure legends or main text, which may have led to misunderstanding regarding data duplication. In consideration of potential reader confusion and in keeping with current standards of academic rigor and transparency, we believe it is appropriate to formally clarify this point. We have now revised all relevant figure legends to explicitly state that the two animal models were established and analyzed concurrently within the same experimental batch and that the control group (Group C) data were intentionally shared across both analyses. These clarifications do not affect the experimental results or the scientific conclusions of the study. It is important to emphasize that the images shown in **Figures 1C**, **4B** are themselves original and accurate; no data have been duplicated or manipulated. The only modification made is to the figure legends, which have now been revised to explicitly state that the two animal models were established and analyzed concurrently within the same experimental batch and that the control group (Group C) data were intentionally shared across both analyses. These clarifications do not affect the experimental results or the scientific conclusions of the study. The corrected captions for **Figures 1**–**6** appear below.

**Figure 2:**
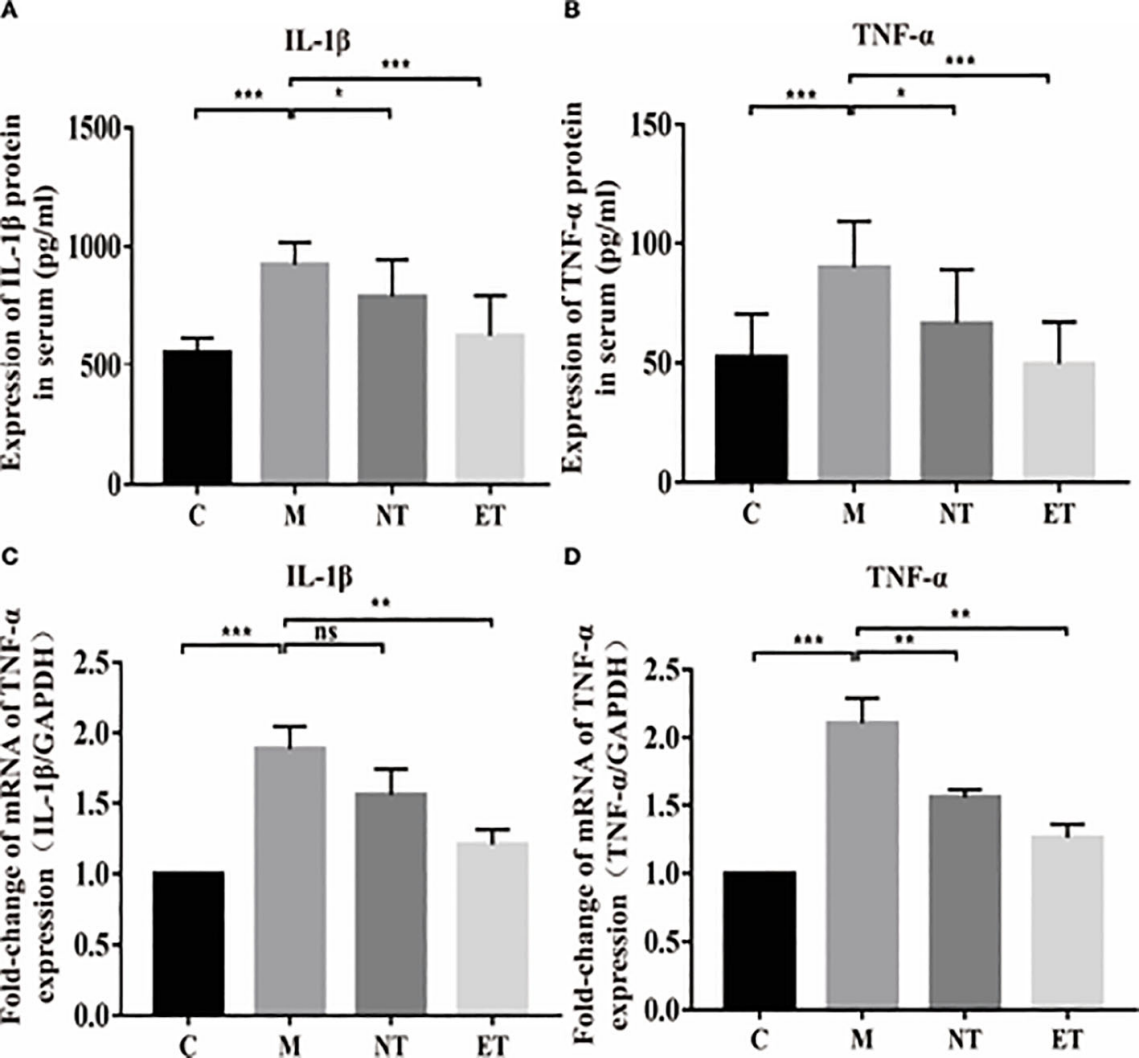
Effects of *L. crispatus*-pMG36e-mCXCL12 on the expression of pro-inflammatory factors at the protein and gene levels in intrauterine adhesion mice. Effect of *L. crispatus*-pMG36e-mCXCL12 on the expression of IL-1β **(A)** and TNF-α **(B)** in the uterine tissue of intrauterine adhesion mice at the protein level. Effect of *L. crispatus*-pMG36e-mCXCL12 on the expression of IL-1β **(C)** and TNF-α **(D)** in the uterine tissue of intrauterine adhesion mice at the gene level. C group, Control group; M group, laparotomy was used to construct a model of intrauterine adhesion; NT group was treated with *L. crispatus* for intrauterine adhesion mice; ET group was treated with *L. crispatus*-pMG36e-mCXCL12 for intrauterine adhesion mice. ns, P > 0.05; *P < 0.05; **P < 0.01; ***P < 0.001. Two animal models (IUA and IUA with diabetes) were established and analyzed concurrently within the same experimental batch and that the same Group C control samples from the same experimental batch were intentionally shared across both models.

**Figure 3:**
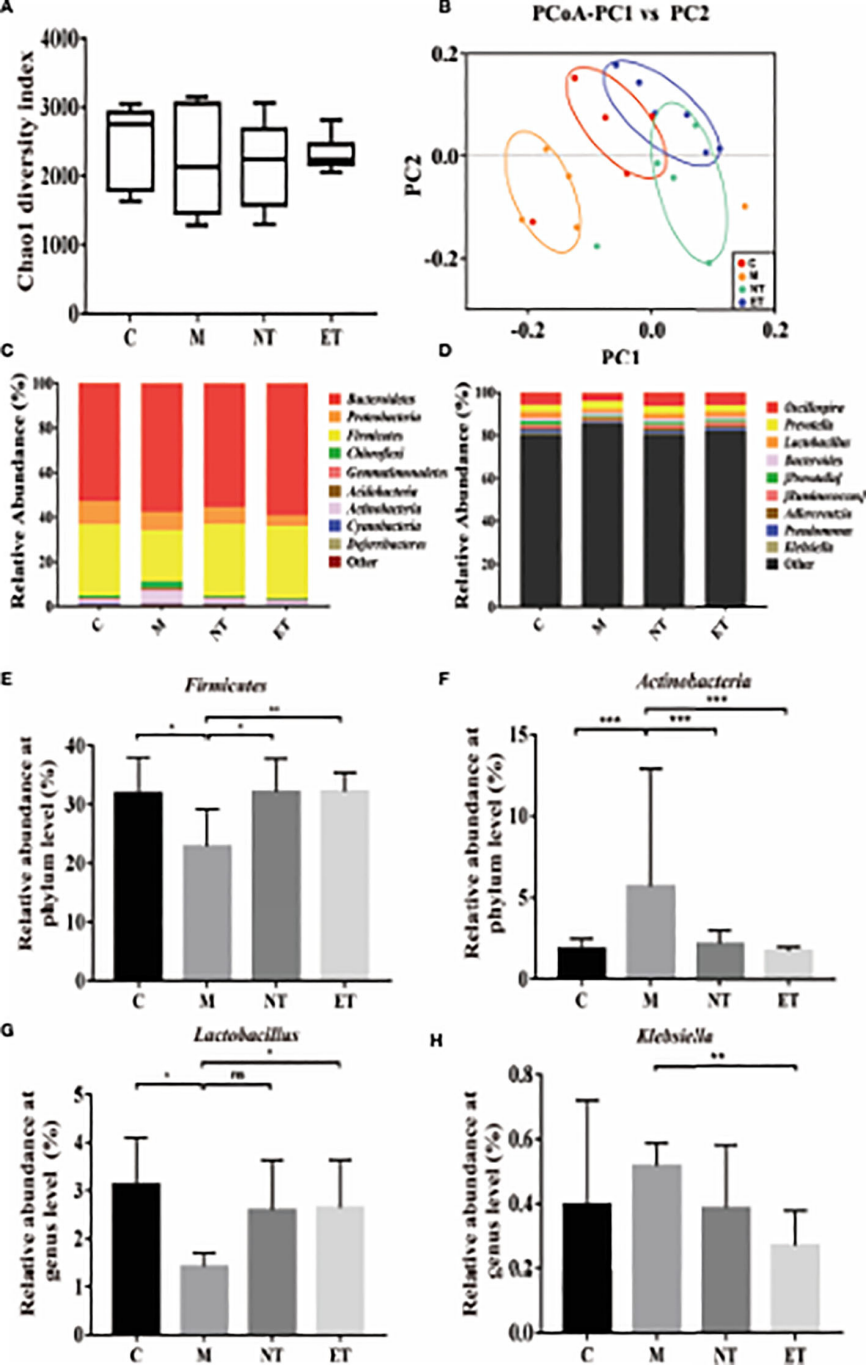
Effects of *L. crispatus*-pMG36e-mCXCL12 on vaginal microbiota in intrauterine adhesion mice. Evaluation of the effect of *L. crispatus*-pMG36e-mCXCL12 on vaginal microbiota of intrauterine adhesion mice using **(A)** the Chao1 Diversity, **(B)** the PCoA of the β diversity index, the relative abundance **(C)** at the phylum level and **(D)** at the genus level. Evaluation of the effect of *L. crispatus*-pMG36e-mCXCL12 on the relative abundances of the phyla **(E)** *Firmicutes*, **(F)** *Actinobacteria* in intrauterine adhesion mice; Evaluation of the effect of *L. crispatus*-pMG36e-mCXCL12 on the relative abundances of the genera **(G)** *Lactobacillus*, **(H)** *Klebsiella* in intrauterine adhesion mice. C group, Control group; M group, laparotomy was used to construct a model of intrauterine adhesion; NT group was treated with *L. crispatus* for intrauterine adhesion mice; ET group was treated with *L. crispatus*-pMG36e-mCXCL12 for intrauterine adhesion mice. ns, P > 0.05; *P < 0.05; **P < 0.01; ***P < 0.001. Two animal models (IUA and IUA with diabetes) were established and analyzed concurrently within the same experimental batch and that the same Group C control samples from the same experimental batch were intentionally shared across both models.

**Figure 4:**
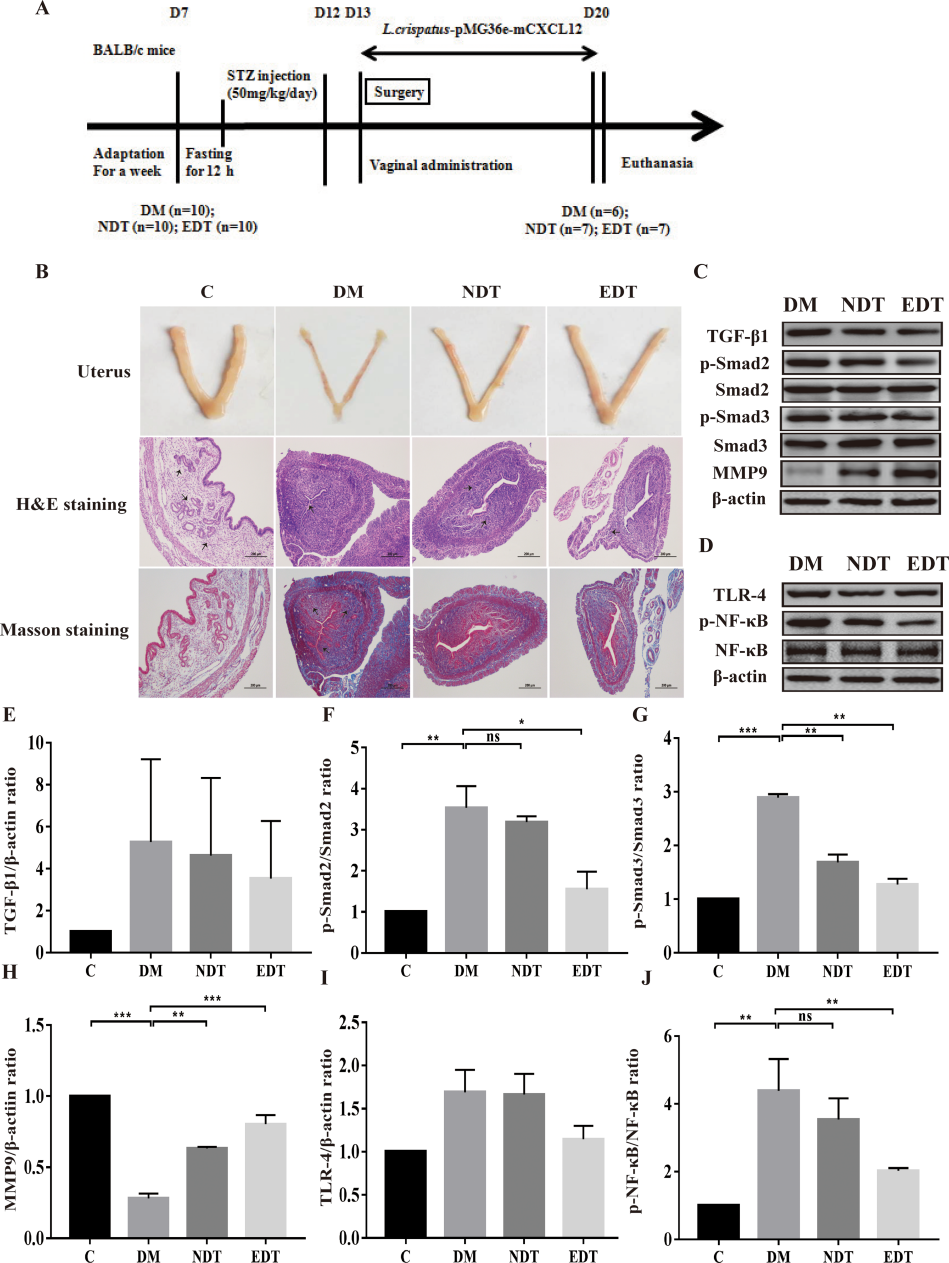
Effect of *L. crispatus*-pMG36e-mCXCL12 on the expression of proteins associated with the TGF-β1/Smads and TLR4/NF-κB signalling pathway in intrauterine adhesion mice with diabetes. **(A)** Experimental scheme to evaluate the effects of *L. crispatus*-pMG36e-mCXCL12 on the prevention of intrauterine adhesion in diabetic mice. **(B)** H&E staining was used to observe uterine inflammation, and Masson staining was used to observe collagen deposition in the uterine tissue of intrauterine adhesion mice with diabetes (magnification: × 100). **(C)** Expression of fibrotic-related proteins in the uterine tissue of intrauterine adhesion mice with diabetes. **(D)** Expression of inflammatory-related proteins in the uterine tissue of intrauterine adhesion mice with diabetes. Effect of *L. crispatus*-pMG36e-mCXCL12 on fibrotic-related TGF-β1 **(E)**, p-Smad2/Smad2 **(F)**, p-Smad3/Smad3 **(G)**, MMP-9 **(H)** proteins in the uterine tissue of intrauterine adhesion mice with diabetes. Effect of *L. crispatus*-pMG36e-mCXCL12 on inflammatory-related TLR4 **(I)**, p-NF-κB/NF-κB **(J)** proteins in the uterine tissue of intrauterine adhesion mice with diabetes. C group, Control group; DM group, laparotomy was used to construct a model of intrauterine adhesion mice with diabetes; NDT group was treated with *L. crispatus* for intrauterine adhesion mice with diabetes; EDT group was treated with *L. crispatus*-pMG36e-mCXCL12 for intrauterine adhesion mice with diabetes. ns, P > 0.05; *P < 0.05; **P < 0.01; ***P < 0.001. Two animal models (IUA and IUA with diabetes) were established and analyzed concurrently within the same experimental batch and that the same Group C control samples from the same experimental batch were intentionally shared across both models.

**Figure 5:**
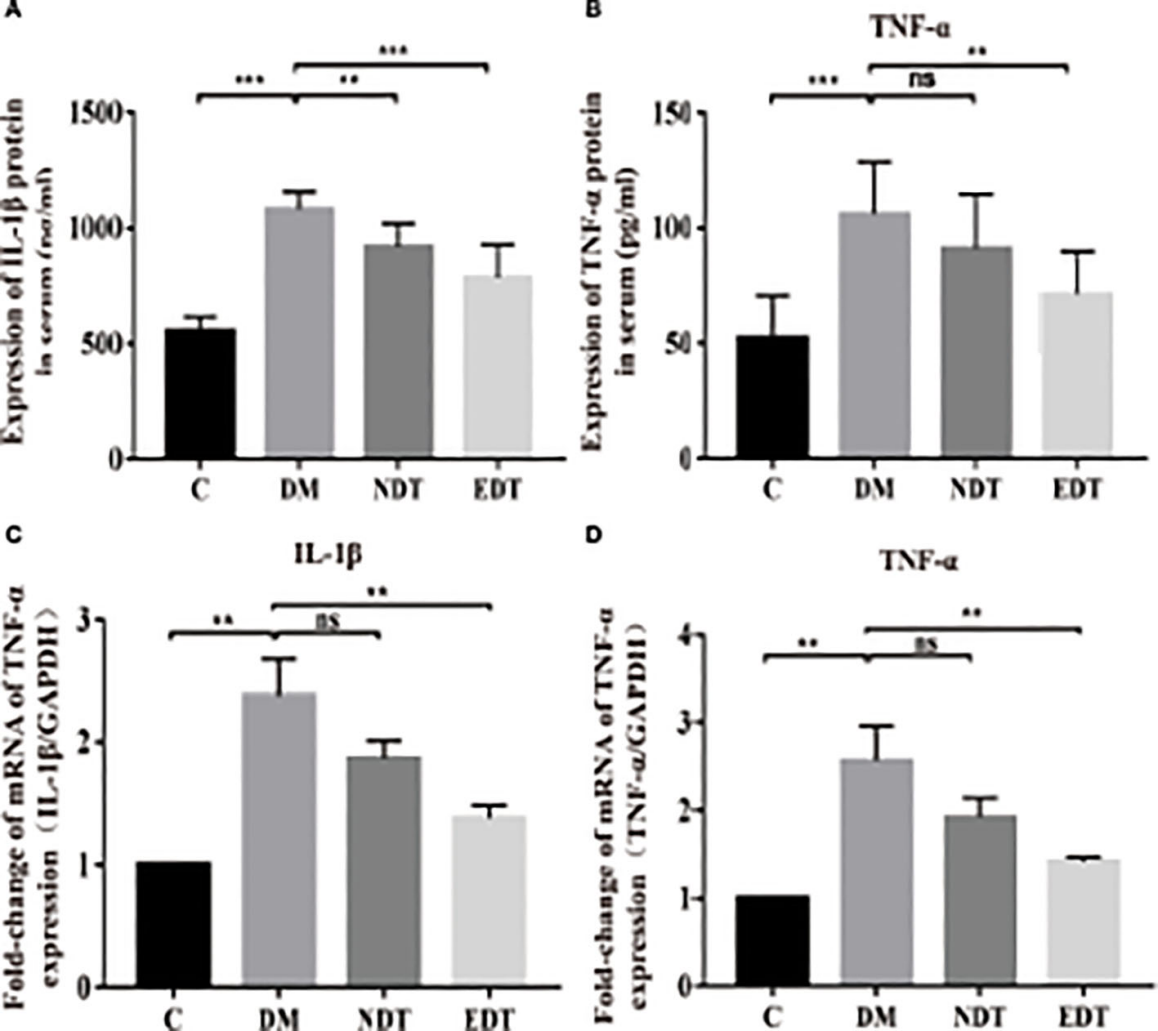
Effects of *L. crispatus*-pMG36e-mCXCL12 on the expression of pro-inflammatory factors at the protein and gene levels in intrauterine adhesion mice with diabetes. Effect of *L. crispatus*-pMG36e-mCXCL12 on the expression of IL-1β **(A)** and TNF-α **(B)** in the uterine tissue of intrauterine adhesion mice with diabetes at the protein level. Effect of *L. crispatus*-pMG36e-mCXCL12 on the expression of IL-1β **(C)** and TNF-α **(D)** in the uterine tissue of intrauterine adhesion mice with diabetes at the gene level. C group, Control group; DM group, laparotomy was used to construct a model of intrauterine adhesion mice with diabetes; NDT group was treated with *L. crispatus* for intrauterine adhesion mice with diabetes; EDT group was treated with *L. crispatus*-pMG36e-mCXCL12 for intrauterine adhesion mice with diabetes. ns, P > 0.05; *P < 0.05; **P < 0.01; ***P < 0.001. Two animal models (IUA and IUA with diabetes) were established and analyzed concurrently within the same experimental batch and that the same Group C control samples from the same experimental batch were intentionally shared across both models.

**Figure 6:**
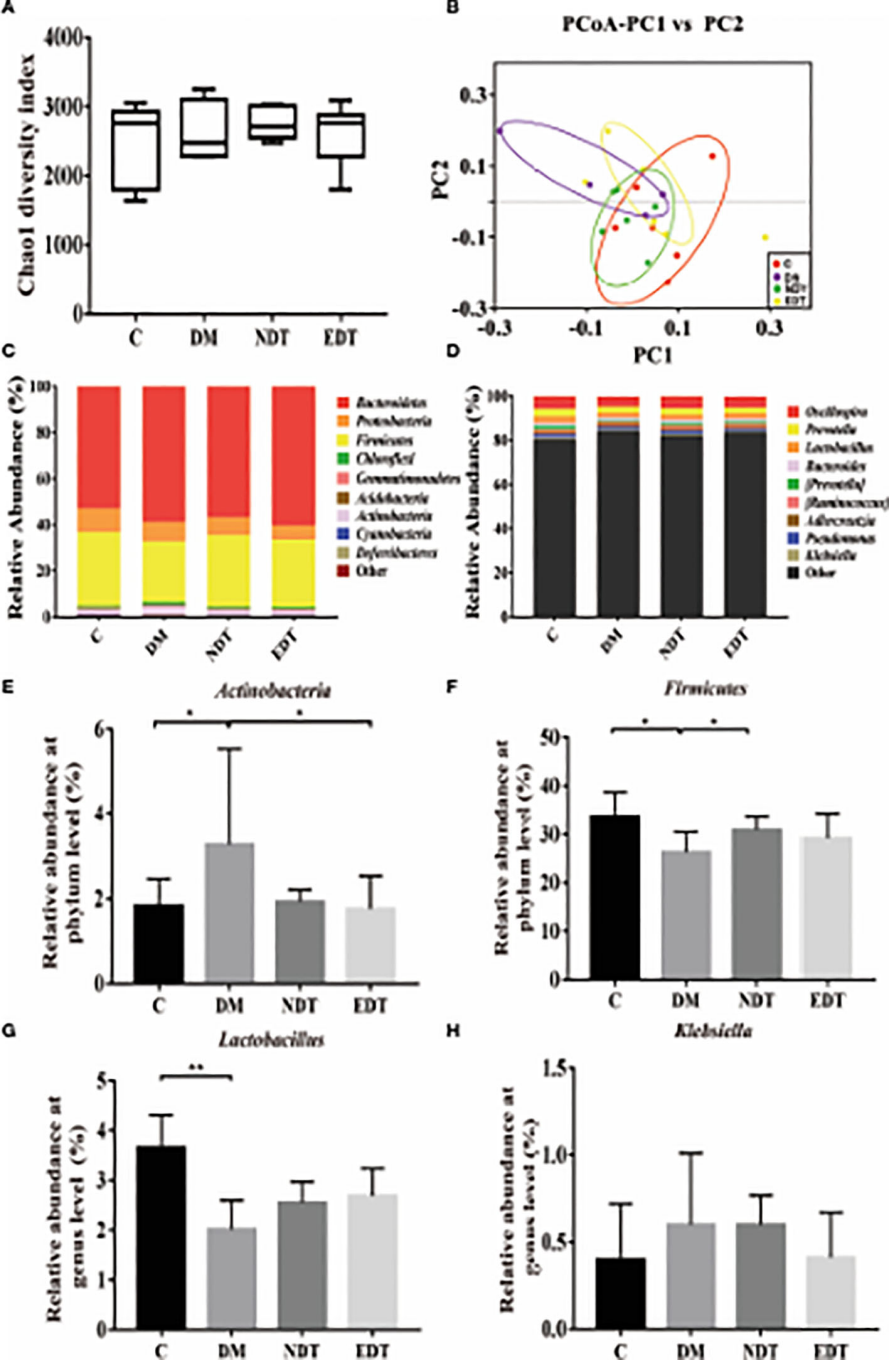
Effects of *L. crispatus*-pMG36e-mCXCL12 on vaginal microbiota in intrauterine adhesion mice with diabetes. Evaluation of the effect of *L. crispatus*-pMG36e-mCXCL12 on vaginal microbiota of intrauterine adhesion mice with diabetes using **(A)** the Chao1 Diversity, **(B)** the PCoA of the β diversity index, the relative abundance **(C)** at the phylum level and **(D)** at the genus level. Evaluation of the effect of *L. crispatus*-pMG36e-mCXCL12 on the relative abundances of the phyla **(E)** *Firmicutes*, **(F)** *Actinobacteria* in intrauterine adhesion mice with diabetes; Evaluation of the effect of *L. crispatus*-pMG36e-mCXCL12 on the relative abundances of the genera **(G)** *Lactobacillus*, **(H)** *Klebsiella* in intrauterine adhesion mice with diabetes. C group, Control group; DM group, laparotomy was used to construct a model of intrauterine adhesion mice with diabetes; NDT group was treated with *L. crispatus* for intrauterine adhesion mice with diabetes; EDT group was treated with *L. crispatus*-pMG36e-mCXCL12 for intrauterine adhesion mice with diabetes. ns, P > 0.05; *P < 0.05; **P < 0.01; ***P < 0.001. Two animal models (IUA and IUA with diabetes) were established and analyzed concurrently within the same experimental batch and that the same Group C control samples from the same experimental batch were intentionally shared across both models.

The original version of this article has been updated.

