# Supplementary material for: Protective Effects of Engineered Lactobacillus crispatus on Intrauterine Adhesions in Mice via Delivering CXCL12
Source: Front Immunol. 2022 Jun 6;13:905876. doi: 10.3389/fimmu.2022.905876 (PMC9207254; doi:10.3389/fimmu.2022.905876)
Supplement: Supplementary file 1 [file Table1.doc]

| Table S1 Sequences of the reverse transcription quantitative polymerase chain reaction primers used. | |
| --- | --- |
| Name | Sequence |
| IL-1β | Forward: 5'-GTGTCTTTCCCGTGGACCTTC-3' Reverse: 5'-TCATCTCGGAGCCTGTAGTGC-3' |
| TNF-α | Forward: 5'-GTGGAACTGGCAGAAGAGGCA-3' Reverse: 5'-AGAGGGAGGCCATTTGGGAAC-3' |
| GAPDH | Forward: 5'-CTCGTGGAG TCTACTGGTGT-3'  Reverse: 5'-GTCATCATACTTGGCAGGTT-3' |
