# Supplementary material for: Protective Effects of Engineered Lactobacillus crispatus on Intrauterine Adhesions in Mice via Delivering CXCL12
Source: Front Immunol. 2022 Jun 6;13:905876. doi: 10.3389/fimmu.2022.905876 (PMC9207254; doi:10.3389/fimmu.2022.905876)
Supplement: Supplementary file 2 [file Table2.docx]

Table S2 Genetic stability evaluation of pMG36e-mCXCL12 recombinant plasmid

| Time (Day) | Genetic stability rate (%) | |
| --- | --- | --- |
|  | Erythromycin (+) | Erythromycin (-) |
| 0 | 100 | 100 |
| 3 | 100 | 100 |
| 6 | 100 | 100 |
| 9 | 100 | 99 |
| 12 | 100 | 98 |
| 15 | 100 | 96 |
| 18 | 100 | 93 |
| 21 | 100 | 91 |
| 24 | 100 | 88 |
| 27 | 100 | 85 |
| 30 | 100 | 83 |

Fig S2. The mCXCL12 in vitro bacterial expression. Levels of mCXCL12 in the supernatant bacteria pellet break supernatant after 24 h of culture as determined by ELISA
